# An Integrated *In Silico* Approach to Design Specific Inhibitors Targeting Human Poly(A)-Specific Ribonuclease

**DOI:** 10.1371/journal.pone.0051113

**Published:** 2012-12-06

**Authors:** Dimitrios Vlachakis, Athanasia Pavlopoulou, Georgia Tsiliki, Dimitri Komiotis, Constantinos Stathopoulos, Nikolaos A. A. Balatsos, Sophia Kossida

**Affiliations:** 1 Bioinformatics and Medical Informatics Laboratory, Biomedical Research Foundation of the Academy of Athens, Athens, Greece; 2 Department of Biochemistry and Biotechnology, University of Thessaly, Larissa, Greece; 3 Department of Biochemistry, School of Medicine, University of Patras, Rio-Patras, Greece; Duke University Medical Center, Duke University, United States of America

## Abstract

Poly(A)-specific ribonuclease (PARN) is an exoribonuclease/deadenylase that degrades 3′-end poly(A) tails in almost all eukaryotic organisms. Much of the biochemical and structural information on PARN comes from the human enzyme. However, the existence of PARN all along the eukaryotic evolutionary ladder requires further and thorough investigation. Although the complete structure of the full-length human PARN, as well as several aspects of the catalytic mechanism still remain elusive, many previous studies indicate that PARN can be used as potent and promising anti-cancer target. In the present study, we attempt to complement the existing structural information on PARN with in-depth bioinformatics analyses, in order to get a hologram of the molecular evolution of PARNs active site. In an effort to draw an outline, which allows specific drug design targeting PARN, an unequivocally specific platform was designed for the development of selective modulators focusing on the unique structural and catalytic features of the enzyme. Extensive phylogenetic analysis based on all the publicly available genomes indicated a broad distribution for PARN across eukaryotic species and revealed structurally important amino acids which could be assigned as potentially strong contributors to the regulation of the catalytic mechanism of PARN. Based on the above, we propose a comprehensive *in silico* model for the PARN’s catalytic mechanism and moreover, we developed a 3D pharmacophore model, which was subsequently used for the introduction of DNP-poly(A) amphipathic substrate analog as a potential inhibitor of PARN. Indeed, biochemical analysis revealed that DNP-poly(A) inhibits PARN competitively. Our approach provides an efficient integrated platform for the rational design of pharmacophore models as well as novel modulators of PARN with therapeutic potential.

## Introduction

The first and often rate-limiting step in eukaryotic mRNA turnover is the shortening of the poly(A) tail [1]–[4]. The process is known as deadenylation and it occurs both in the nucleus and in the cytoplasm. In the nucleus it restricts newly added poly(A) tails to their appropriate lengths. In the cytoplasm, deadenylation either decreases the total mRNA levels and regulates the expression levels of specific mRNAs, or modulates the length of the poly(A) tail. Deadenylation is catalyzed by a family of specific ribonucleases, known as deadenylases [4]–[6]. Among these, poly(A)-specific ribonuclease (PARN) has been involved in key biological processes, such as development, cell cycle progression, DNA damage response and cancer. PARN is conserved in many eukaryotes from yeast and plants to humans. PARN homologs are found in *Schizosaccharomyces pombe* (fission yeast) and *Anopheles gambiae* (mosquito), but they are notably absent from *Saccharomyces cerevisiae* and *Drosophila melanogaster* [5]–[7], suggesting that they are not required by all eukaryotes [5]. Structural and biochemical studies revealed that PARN is homodimeric and the active site consists of four acidic amino acids Asp28, Glu30, Asp292, and Asp382, which are believed to coordinate the catalytically important divalent metal ions [8]–[9]. Furthermore, the residue His377, which is conserved in PARN, has also been proposed to be essential for catalytic activity, thus classifying PARN as a DEDDh nuclease [9], named after the five conserved catalytic amino acid residues. The structure of PARN is composed of at least three functional domains: the catalytic nuclease domain, and two RNA binding domains: the R3H domain and the RNA binding domain or RNA recognition motif (RRM) [9]–[10] which have been suggested to contribute to the catalytic activity of the enzyme [9]–[12]. The RRM is a unique, multifunctional domain that is responsible for molecular recognition of the 5′ cap structure [13]. The latter is perhaps the most characteristic feature of PARN that distinguishes it from all the other known deadenylases. Cap-binding has been reported to significantly contribute to the processivity of the enzyme. Apart 5′-cap, PARN activity is regulated by natural nucleotides [14]–[16] and by several protein factors. The latter include the cytoplasmic poly(A)-binding protein (PABPC) [17], the eukaryotic initiation factor 4E (eIF4E) [18] and the nuclear cap-binding complex (CBC) that negatively regulate PARN [19], while RHAU helicase [20] and AU-rich element (ARE) - binding proteins, including TTP and KSRP are positive regulators [21]–[23]. PARN activity is also regulated by factors that bind cytoplasmic polyadenylation elements (CPEs) including CPE-binding protein (CPEB) and the atypical Gld2 poly(A) polymerase [24], [25]. Finally, PARN has been shown to be a target of synthetic nucleoside analogs with anticancer and antiviral potential. These analogs inhibit PARN activity in a competitive mode [26], [27]. Furthemore, PARN mRNA and protein expression levels are elevated in acute leukemias [28]. These observations suggest that that enzyme may be a promising biomarker and a target for drug design [28].

Herein, we present a PARN-specific 3D pharmacophore model both for *de novo* design and virtual screening of selective inhibitors. For the design of the pharmacophore model, we initially used an in-depth phylogenetic analysis of PARN across species, which identified structurally conserved residues, important for the catalytic activity of the enzyme. Using a series of computer-aided molecular simulations, supported by statistical structure-activity correlations of our previously reported nucleoside analogs that inhibit PARN, we established a combined complex-based 3D pharmacophore model. We applied our *in silico* model to predict the effect of the amphipathic DNP-poly(A) substrate as a novel PARN-interacting molecule, which was then confirmed to efficiently inhibit the enzyme by kinetic assays.

## Results and Discussion

### Phylogenetic Analysis of PARN

The complex-based 3D pharmacophore for the specific drug design of novel PARN inhibitors was based on a) a comprehensive phylogenetic analysis to identify evolutionary invariant amino acids across species, b) *in silico* conformational evaluation of these residues in the context of the overall structure and the catalytic mechanism, and c) substrate preferences and results from previous compounds that inhibit PARN efficiently.

**Figure 1:**
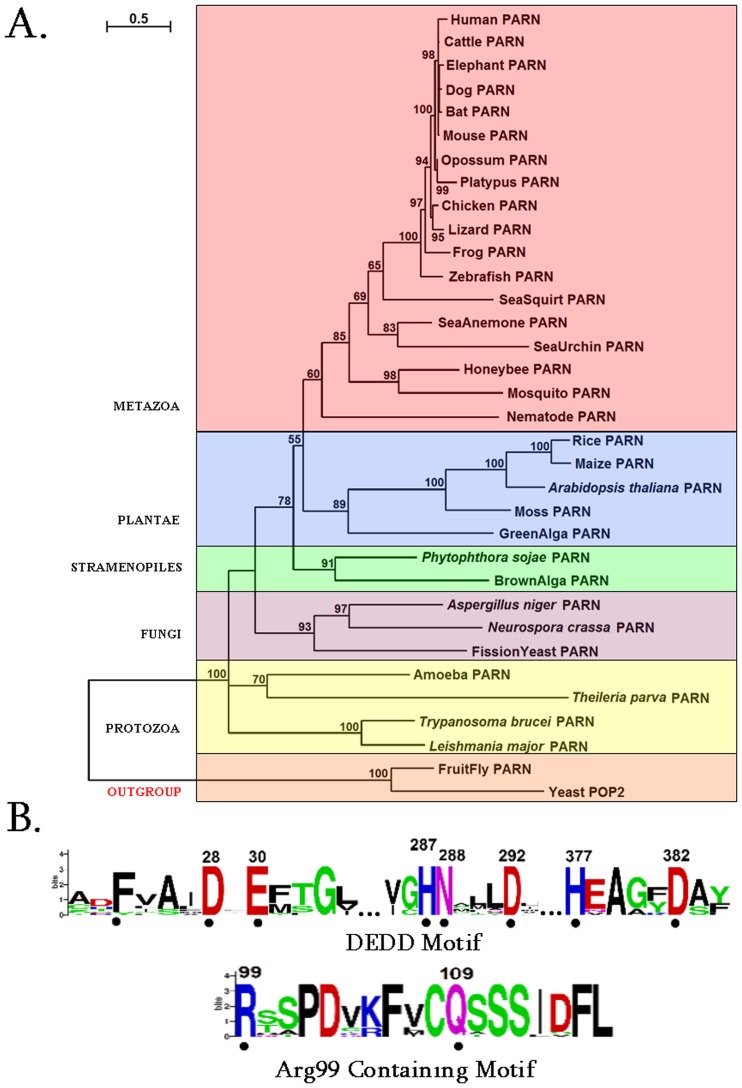
PARN phylogenetic analysis and sequence motifs. (A) Phylogenetic tree of PARN proteins. Colored boxes identify different eukaryotic groups. Bootstrap values (>50%) are shown at the nodes. The length of the tree branch reflects evolutionary distance. The scale bar at the upper left represents evolutionary distance of 0.5 amino acids per position.(B) Sequence logo of the motifs identified in PARN protein sequences. The amino acid residue numbers (according to human PARN numbering) are indicated at the top. The height of each letter is proportional to the frequency of the corresponding residue at that position, and the letters are ordered so the most frequent is on the top. The invariant residues are indicated with dots.

Firstly we performed a comprehensive phylogentic analysis of PARN. Collectively, 32 homologous PARN protein sequences were identified in the genomes of species, which represent diverse eukaryotic taxonomic divisions (according to the NCBI taxonomy database) [29] (Table S1). Therefore, PARN exhibits a broad phylogenetic distribution, ranging from protozoa to metazoa (Fig. 1A).

In agreement with previous reports, PARN homologs were not found in the arthropod *Drosophila melanogaster* (fruit fly) and the fungus *Saccharomyces cerevisiae* (yeast) [5]–[7]. Alternative metabolic pathways may exist in these two organisms for poly(A) degradation, as in the case for amino acid starvation control [30]. However, putative PARN homologous sequences were detected in other arthopods and fungi (Table S1).

Based on the reconstructed phylogenetic tree in Fig. 1A, PARN sequences from different eukaryotic groups form separate monophyletic clades, supported by relatively high bootstrap values. The Drosophila and yeast POP2 [31], [32] sequences were selected as outgroups (Fig. 1A). Even though POP2 does not belong to the DEDDh subfamily of exonucleases and shares only 17% sequence identity with PARN, the structure of the core nuclease domains of both enzymes are very similar [9]. The major difference between PARN and POP2 is PARN’s 5′-cap binding specificity, which may not be required in *Drosophila melanogaster* and *Saccharomyces cerevisiae*.

Further, protein motifs were derived from the multiple alignments of PARN amino acid sequences (Fig. 1B). Apart from the conserved catalytic motif (Asp28, Glu30, Asp292 and Asp382), a second motif containing the invariant Arg99 and Gln109 residues was detected only in metazoa (Fig. 1B). Upon careful examination of the primary amino acid sequence of other species besides Metazoa, we found that in the neighboring Arg99 region either there are Arg residues, or Arg has been replaced by the fellow polar residue Lys. The observation that Arg99 is evolutionary invariant only in metazoa (Fig. 1B) prompted us to investigate its structural conservation across non-metazoa species by homology modeling. Indicatively, the corresponding sequences for PARN from *Arabidopsis thaliana* and *Trypanosoma brucei* were aligned against human PARN, which was used as template. Careful inspection of the final homology models, after energy minimization, revealed that the spatial coordinates of human PARN Arg99 were identical to the residue Arg89 of PARN from *Arabidopsis thaliana* (Fig. S1). On the contrary, the homology model of *Trypanosoma brucei* completely lacks the Arg99-corresponding residue in its 3D structure of PARN.

Collectively, PARN was found in all eukaryotes, but the arthropod *Drosophila melanogaster* (fruit fly) and the fungus *Saccharomyces cerevisiae* (yeast). Moreover, a series of invariant residues were identified, which were subsequently structurally investigated for any possible involvement in the catalytic regulation of PARN.

### Arg99 and Gln109 are Involved in the Regulation of Catalysis

**Figure 2:**
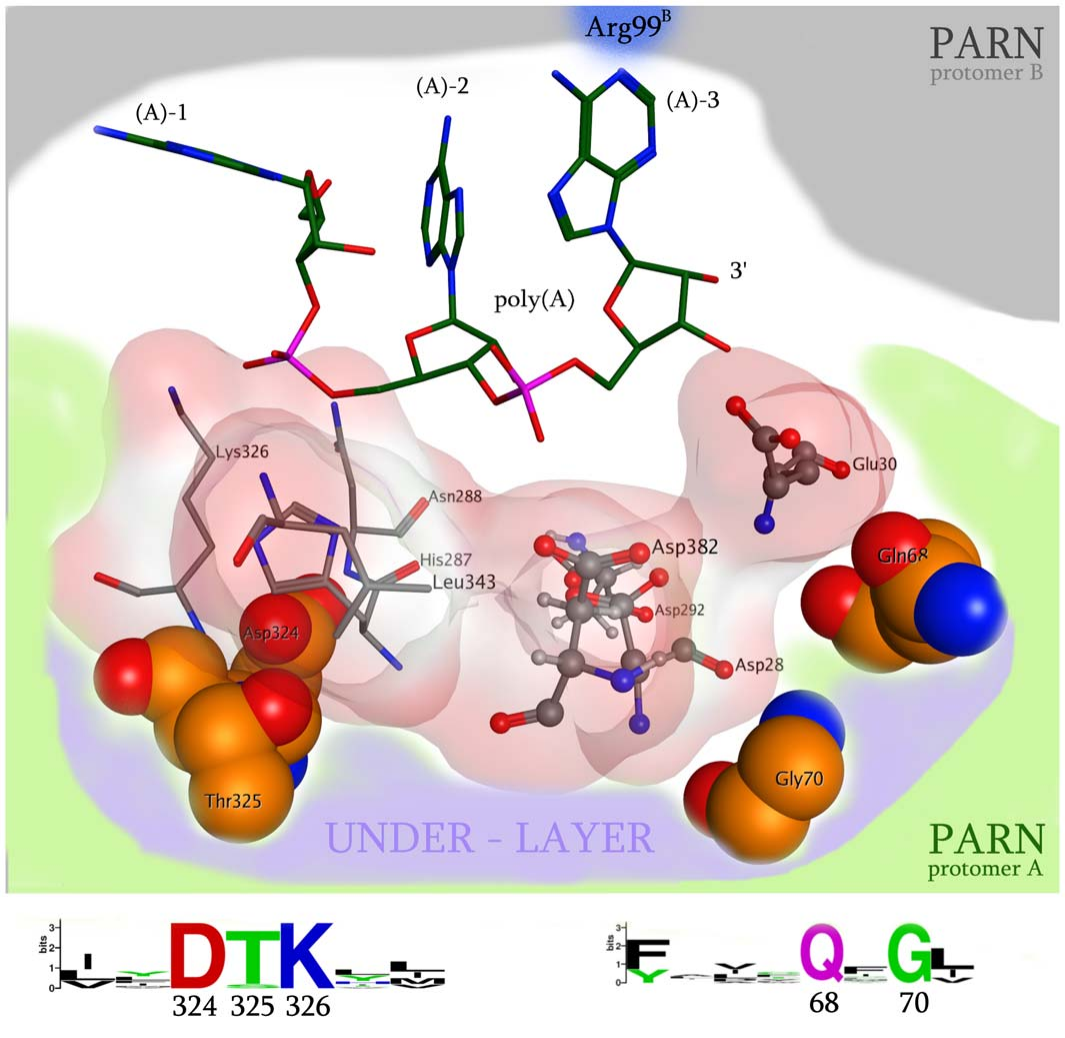
A representation of the 3D organization of the catalytic site of PARN. The RNA interacting and structurally conserved residues (Asp324, Thr325, Gly70, Gln68, Leu343, Asn288, Lys326) are shown in an electrostatic cloud, whereas the four evolutionary invariant amino acids that conformationally support the catalytic residues are shown in specefill representation (labeled as under-layer, Asp324, Thr325, Gln68, Gly70). The invariant residues that were detected in the PARN protein motifs by our phylogenetic analysis are showing below the 3D structure.

**Figure 3:**
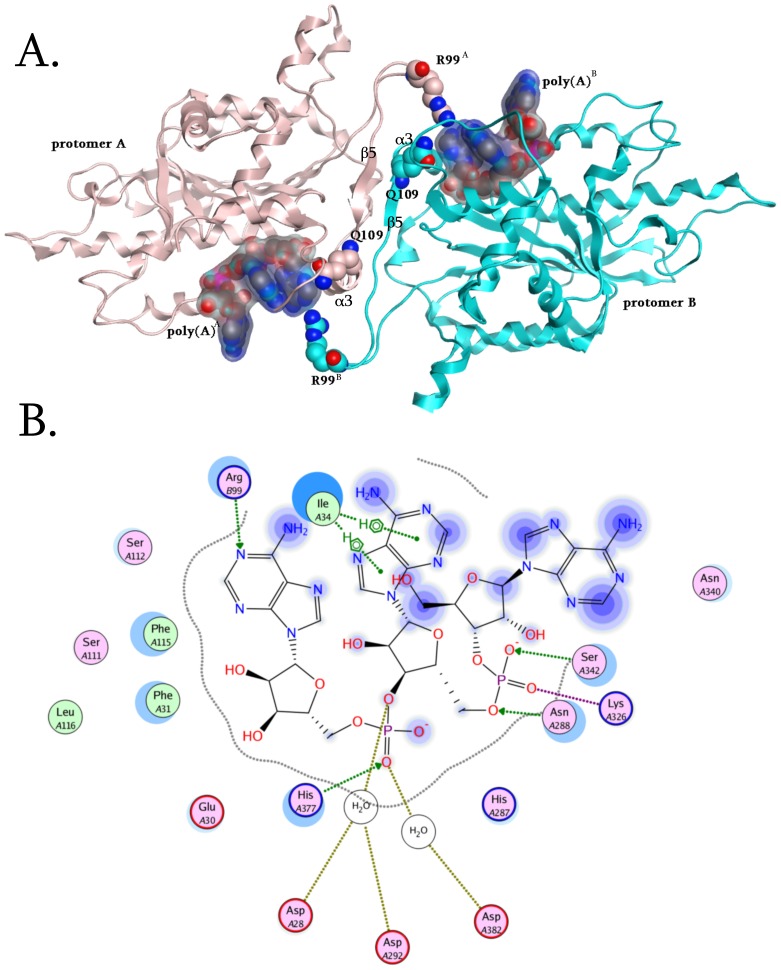
The role of Arg99 in the catalytic mechanism of PARN. (A) PARN - Poly(A) interactions have been calculated for both active sites. The Arg99 residues have been highlighted while they are H-bonding with the base moiety of the first poly(A) nucleotide. (B) The interaction map of poly(A) and the catalytic site of human PARN, showing the water mediated bridges of the Aspartic residue attacking the first phosphodiesteric bond, and the vital contribution of the invariant, structurally conserved His377 residue.

Based on the phylogenetic analysis, we further focus on the possible roles of the invariant Arg99 and Gln109 residues. PARN is a homodimeric enzyme where each monomer harbors an identical catalytic active site (Fig. 2), and at least in humans, PARN is only active in its dimeric form [9]. Structural superposition of the two monomers and the two corresponding poly(A) oligonucleotides reveal only minor deviations (max Ca RMSD <2 Å). Our *in silico* structural analysis revealed that Arg99 of monomer A (Arg99^A^) is contributed by the complementary monomer during catalysis in a symmetric fashion. In particular Arg99^A^ extends into the catalytic site of chain B, as does Arg99^B^ to the catalytic site of chain A. These arginine residues establish hydrogen bonding with the adenine base of the last 3′ adenosine nucleoside of the poly(A) chain. The hydrogen bond is achieved by electron transfer between the -NH_2_ group (donor) of the arginine and the –N = group (acceptor) of the six-member ring of adenine (Fig. 3A–B). The essential contribution of the Arg99 residue was also confirmed by mutation studies on α3 helix of PARN, which is a conformational flexible loop on the counterpart monomer, and supports Arg99 in the proximity of the catalytic region [9]. MDs of just one monomer of PARN, indicated that in the absence of the α3 counterpart helix, the loop carrying the Arg99 residue is not structurally supported anymore and therefore moved away from the active site having lost completely its interactions with the poly(A) oligonucleotide (Fig. 3A).

Moreover, Ile34 establishes hydrophobic interactions with the conjugated adenine rings of the second nucleotide, thus tethering it in the conformational space of the active site (Fig. 3). The hydrogen bonding interaction between the adenine ring of the first nucleoside and Arg99 of the complementary monomer is much stronger than the hydrophobic interactions established between the corresponding conjugated rings of the second base and Ile34. Subsequently, the involvement of the penultimate scissile bond in the catalytic mechanism was investigated. It was found that hydrogen bonding interactions were established between Asn288, Lys326 and Ser342 residues of PARN and the second scissile bond of the poly(A) substrate. Interestingly, our phylogenetic analysis determined that both Asn288 and Lys326 are invariant residues across species, ranging from protozoa to metazoa. Even though the catalytic function of these residues remains unclear, this is an important finding in itself taking into account that they are both evolutionary conserved, which makes them key pharmacological targets. In any case, nucleotides two and three were both less defined, since they were not rigidly fixated in the 3D conformational space of PARN’s active site. This is supported by crystal structure of PARN, where only the first poly(A) nucleotide was stable enough to return a well defined electron density^9^.

The previous observations suggest that having suppressed the potential degrees of movement for the first two nucleotides by interactions with their bases, PARN can precisely coordinate the positioning of the scissile bond towards the catalytic triad (Asp28, Asp292, Asp382). The optimal catalytic positioning of the scissile bond is directed by His377, which hydrogen bonds to the –P = O oxygen of the phosphodiesteric bond. Consequently, since His377 and Arg99 establish hydrogen bonds with the first nucleotide from different directions, they play a very crucial role in its three-dimensional stabilization and positioning in the catalytic site of PARN (Fig. 3B). This is in agreement with the reported observation that in the crystal structure of PARN, only the first poly(A) nucleotide was stable enough to return a well defined electron density [9].

Previous work has revealed that the β5 strand of one protomer of PARN forms an antiparallel b-sheet with its counterpart from the other protomer. This orientation allows the side chains of conserved residues Phe93, Cys108, Phe106, Ile113, Phe123 and Phe127 from one protomer form extensive hydrophobic interactions with the same set of residues from the other one. Among these, Phe123 is an invariant residue in PARN across species, which when mutated to alanine led to loss of activity [9]. Conclusively, Arg99 may represent another important residue as it links the two monomers, it contributes to overall stability, and directs the substrate to an optimal position for the cleavage reaction.

The other invariant residue across species that was identified from the phylogenetic analysis, Gln109, is located in the two antiparallel β5 strands of the homodimeric interface region. To understand the role of Gln109 we performed the Gln109Ala and Gln109Trp *in silico* mutations. The Gln109Ala mutation revealed a significant loss in the packing and association of the two β5 strands (4 fold energetic loss in packing and association), whereas the Gln109Trp mutation constantly failed, since the bulkier Trp residue could not be accommodated in the homodimerization interface region of PARN (Fig. 3A). It is also evident that the bulky side chain of Gln109 defines the shape and the size of the catalytic pocket that is available to accommodate the poly(A) substrate.

### Ιnsights into PARN’s Nuclease Domain and Interdimeric Interface

We performed structural molecular modeling study of the nuclease domain of PARN. The *in silico* analysis revealed a series of previously unreported amino acids, which are essential for the function of the enzyme. A conformational under-layer in its catalytic site is shaped by Gly70, Gln68, Asp324 and Thr325 residues (Fig. 2); Gly70 and Gln68 provide structural support for Asp28 and Glu30 catalytic residues, while Asp324 and Thr325 support the poly(A)-interacting Lys326 and His287 residues [9]. The functional role of the PARN under-layer catalytic site amino acids was consistent with the phylogenetic analysis of PARN, where Gly70, Gln68, Asp324 and Thr325 residues were found to be invariant across species. Additionally, *in silico* mutagenesis studies followed by molecular dynamics simulations (MDs), to each one of the Asp324, Thr325, Gly70 and Gln68 amino acids produced structural conformational changes in the relative positioning of the supported catalytic residues. More specifically, *in silico* mutation of residues Asp324 and Thr325 to either Alanine (Asp324Ala and Thr325Ala) or to the bulkier Phenylalanine residue (Asp324Phe and Thr325Phe) resulted in loss of the hydrogen bonds between the Lys326 and the Asn288 amino acids with the second scissile bond of the poly(A) substrate (Fig. 3B). Substitution of Gly70 to either Alanine or Phenylalanine amino acid resulted in the shifting of the three Aspartic residues towards the poly(A) substrate, which consequently pushed the latter away from the catalytic site, losing the Arg99 hydrogen bonding. Finally, mutating the Gln68 residue to either Alanine or Phenylalanine resulted in a slight rearrangement of the 3D positioning of the Glu30 residue that led to the tilting of the whole poly(A) substrate and the complete loss of its hydrogen bonding interactions with the catalytic site of PARN. These findings suggest that an evolutionary conserved and highly sophisticated under-layer structure in the catalytic site of PARN is essential for the function of the enzyme (Fig. 2).

Furthermore, it was observed that although the catalytic triad was in very close proximity to the scissile bond, it did not seem to directly interact with it [9]. In depth examination of the active site revealed a smaller cavity within the active site, which in the original X-ray structure coordinate file (RCSB entry: 2A1R) accommodates two water molecules. A MD simulation was set in the presence of the crystallographic waters, and concluded that two water molecules had occupied the small pocket in the active site, now linking Asp28 and Asp292 *via* a H-O-H bridge to the -P–O group of the scissile bond, whereas Asp382 now interacted with via a water mediated bridge with the –P = O group as His377 amino acid (Fig. 3B). This pattern has been observed in many phosphate hydrolyzing enzymes. Namely, in the crystal structure of T7 helicase water molecules occupy the 3D space that divalent metal ions are expected to bind [33]. Strikingly, in the crystallographic structure of the latter the His465 residue acts as γ-phosphate sensor that directs conformational changes in the active site, in a similar fashion to the His377 residue of PARN. Furthermore, in the ATP catalytic site of T7 helicase the only contribution from the neighboring subunit is Arg522, which is analogous to the Arg99 amino acid of PARN and also behaves in a fashion similar to the arginine finger of the Ras GTPase activating proteins [34].

### Insights into Substrate Preference of PARN

The preference of PARN for poly(A) as substrate has been extensively investigated by biochemical assays using all varieties of trinucleotide substrates [35]. As this is important for the design of the pharmacophore, we wished to correlate our *in silico* observations with crystallographic and biochemical data. To this end, a series of poly(U), poly(G) and poly(C) oligonucleotide substrates were subjected to MD simulations using the structure of human PARN (Fig. S2). In the case of poly(U), it was found that the pyrimidine ring of uracil is not long enough to interact with the Arg99 residue of the neighboring monomer of PARN. However, even though a crucial bond is lost, the poly(U) molecule still interacts with the catalytic Glu30, which stabilizes the two hydroxyl groups of the sugar moiety of the first nucleosides, so that His377 can interact with the first scissile bond [Fig. S2, poly(U)]. Accordingly, the penultimate phosphodiesteric bond interacts with the evolutionary invariant Lys326 and Leu343 residues, which position the poly(U) oligonucleotide in space in a pattern similar to that of poly(A). That may explain the reduced (10-fold) activity of poly(U) when compared to poly(A) [35]. On the other hand, while the cytosine bases in poly(C) are stereo-chemically similar and of same length to the purine poly(A) chains, they do not establish hydrogen bonding interactions with the Arg99 amino acid. According to *in silico* analysis the base moiety of the second nucleoside is stabilized by weaker hydrophobic interactions with Ile34, while the -NH_2_ group of the same nucleoside establishes strong H-bonding interactions with Val40 residue. These interactions result in a slight tilt of the axis of the nucleoside [Fig. S2, poly(C)]. Moreover the Asn340 residue establishes H-bonding interactions with the N group of the five-member ring of the first nucleoside. The latter two H-bonds combined result in a poly(C) conformation that is incapable of interacting with Arg99 residue of PARN monomer B. The loss of nucleoside coordination makes the interaction with the catalytic triad and the His377 amino acid impossible and results to loss of activity for PARN. Finally, the poly(G) chain produced the smaller number of interactions with the active site of PARN, upon the MDs. The Phe31 residue H-bonded to the hydroxyl group of the sugar moiety of the first adenosine nucleoside, which resulted in the slight shifting of the first phosphodiesteric bond away from the His377 residue and the catalytic aspartic acids [Fig. S2, poly(G)].

To summarize, our 3D modelling study of the catalytic site of the human PARN, successfully confirmed the natural preference of this enzyme for poly(A) substrates as it has been observed by *in* *vitro* studies, based on a series of biophysical electrostatic and hydrophobic interactions. A model consisting of a series of structurally and conserved aminoacids has been constructed to visualize the poly(A) specificity, which also complies with the reduced preference of PARN for poly(U) substrates.

### 3D Pharmacophore Elucidation and the DNP-poly(A) Substrate

3D Pharmacophore design methods take into account both the three-dimensional structures and binding modes of receptors and inhibitors, in order to identify regions that are favorable or not for a specific receptor-inhibitor interaction [36]–[39]. The description of the receptor-inhibitor interaction pattern is determined by a correlation between the characteristic properties of the inhibitors and their effect on enzymatic activity [40]–[42].

The pharmacophore for PARN-specific compounds was based on a custom designed statistical analysis of structure-activity correlation patterns (see Text S1, Fig. S3), structural information from the catalytic site, and substrate preferences, taking also into account all steric and electronic features that are necessary to ensure optimal non-covalent interactions with the enzyme. The pharmacophoric features investigated, included positively or negatively ionized regions, hydrogen bond donors and acceptors, aromatic regions and hydrophobic areas.

**Figure 4:**
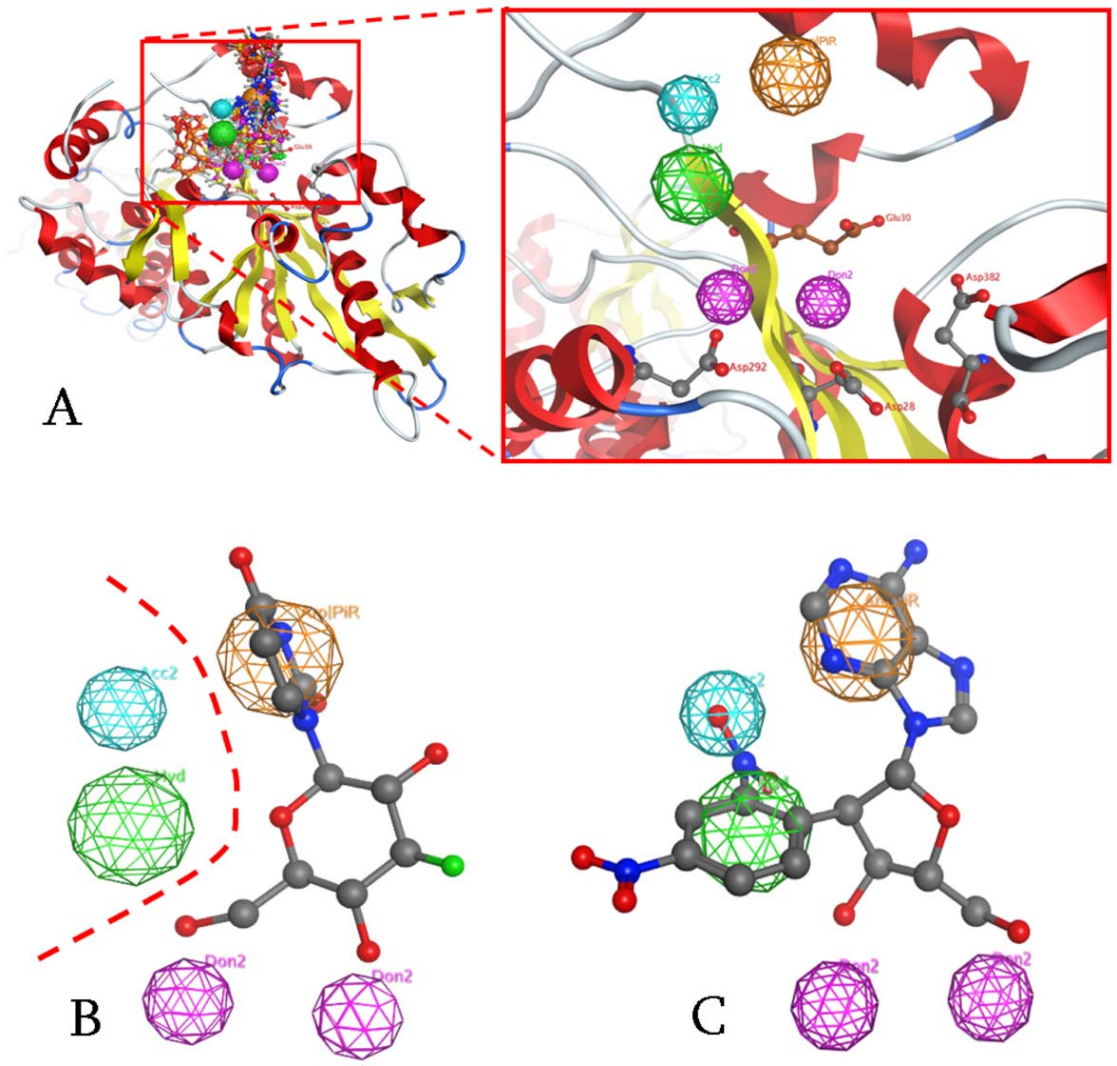
The Pharmacophore proposed for the catalytic site of PARN. (A) All known inhibitors were used to elucidate the consensus PARN Pharmacophore. The three Aspartic acid amino acids of the catalytic triad (Asp28, 292,382) and the Glutamic acid (Glu30) are shown in ball and stick representation. Purple and blue color correspond to electron donating and accepting groups, orange to aromatic moieties and green to hydrophobic interactions. (B) Our proposed pharmacophore is in accordance with our most active compound (U1) for PARN. In contrast, U2 and FU2 compounds are completely inactive, since they are missing the A electron donating position. (C) The DNP-poly(A) compound was identified as a strong *in silico* candidate compound that satisfied all pharmacophore 3D annotation points.

Concerning previously described structure-activity correlation patterns, several nucleoside compounds with inhibitory effect on PARN were used in their *in silico* docked conformations [26]–[27]. Compounds were grouped in two clusters as suggested by our statistical and structural analysis (Table S5 and Table S6): the adenosine-based (A1, A2, A3, A4, A5, A6, A7), and the uracil-, cytosine- and thymidine-based (U1, FU1, U2, FU2, C2, C6, T1, T2). The final pharmacophore was the result of the overlaying of two different pharmacophores that were then reduced to their shared features. In this way only the set of interactions common between the two different pharmacophores were retained. Our complex-based pharmacophore used a query set that represented a set of receptor-inhibitor interaction fingerprints, which were in the form of docked PARN-inhibitor complexes. Firstly, there should be two electron-donating groups (Fig. 4A, purple color) in the proximity of the catalytic triad aspartic acids (Asp28, 292, 382). More precisely, the first electron-donating Pharmacophoric Annotation Point (PAP) would interact with the Asp282 amino acid, whereas the second electron donating PAP with both Asp28 and Asp382 residues. Both electron-donating regions indicate a particular property of the inhibitor and are not necessarily confined to a specific chemical structure. The same PAP represents a variety of chemical groups that share similar properties. Moreover, those two interaction sites may not strictly represent hydrogen bonds, but water or ion mediated bridges, since the distance from the catalytic aspartic acids varies between 4–6 Å. Also, the base region of the nucleoside compounds should be occupied by a large conjugated set of one or two aromatic rings (Fig. 4A, orange color). However the most important factor of the aromatic PAP was the optimal positioning of this group in the 3D conformational space of the active site of PARN, rather than the amount of conjugation in the base moiety. Fig. 4B displays our most potent nucleoside analog inhibitor, U1 with a *K*_i_ of 19 μΜ, in total compliance with the pharmacophore. Interestingly, the complex-based pharmacophore elucidation process identified two more PAP regions in the catalytic site of PARN (Fig. 4B, dotted line). Namely, based on the nature and type of the amino acids that reside in the catalytic site of PARN, a hydrophobic and a hydrogen acceptor region were suggested.

According to our *in silico* prediction model, a potent candidate inhibitor of PARN should satisfy all of the previously described pharmacophoric features. Therefore, using high-throughput virtual screening techniques (HTVS), the NCI compound database was screened for compounds that match the criteria set by the pharmacophore model. The highest ranking compound was found to be the DNP-adenosine, or DNP-(A) nucleoside, which fitted accurately our model in its estimated bioactive conformation (Fig. 4C).

The DNP-(A) analog and the successive DNP-poly(A) polymer constitute a very promising agent with enhanced drug-likeness potential, when compared to adenosine nucleotides [43]. The polymer of DNP-(A) was constructed based on the poly(A) structure co-crystallized in the active site of the human PARN enzyme (2A1R). The fact that an adenine based inhibitor substrate was selected was quite encouraging, given PARN’s increased affinity for adenine-based oligonucleotides. However, the latter are too polar to cross the cell membranes and therefore cannot be used as a platform for the putative design for potential PARN inhibitors. On the contrary, the DNP moiety of the DNP-poly(A) substrate contributes amphipathically to the molecule which enables it to be more membrane-permeable compared to poly(A) chains [43]. Macromolecular therapeutic agents bear great potential as drug candidates but often fail to cross biological membranes. The DNP-poly(A) substrate was found to be capable of transporting rapidly and freely through cellular membranes and viruses, while poly(A) oligonucleotides could not [43]. Furthermore DNP-poly(A) was found to be both nuclease-resistant and to have strong antiviral and anti-reverse transcriptase properties [43]. The previous support the hypothesis that DNP-poly(A) is a compound far more versatile than poly(A), since it provides the platform and the drug-likeness required for the rational design of anti-PARN agents.

The *in silico* prediction of the inhibitory activity of DNP-poly(A) is based primarily on a direct comparison of the latter to poly(A) polymers. Therefore, a dihedral energy plot was constructed for the poly(A) monomer (adenine) and for the DNP-poly(A) monomer (Fig. S4A–B). By calculating the dihedral energy plot of the rotatable bond linking the sugar to the base moiety it was determined that the rotation energy for adenosine varies between 0–2,5 Kcal/mole whereas the corresponding energy for NNP-(A) varies from 0–11,5 Kcal/mole (Fig. S4D), which meant that the DNP moiety exhibits steric hindrance with the base of the DNP-(A) monomer for a set of given angles.

The maneuverability of the poly(A) substrate from the crystal structure of PARN was then compared to a custom made DNP-poly(A) molecule of the same length in the active site of PARN. It is clear that the dihedral rotating angles of the DNP-poly(A) chain are much more constricted than the poly(A) chain. The calculation was repeated *in vacuo* in the absence of PARN, where the DNP-poly(A) molecule appeared more rigid than poly(A). More specifically, the DNP moiety of the first nucleotide establishes pi-stacking hydrophobic interactions with the Phe31 residue, which does not engage in any form of interaction with the poly(A) substrate (Fig. S5). Notably, the two hydrogen bonds between the first base of poly(A) and the Arg99 and His377 residues have been conserved with the DNP-poly(A) substrate too. Conclusively, the role of this extra pi-stacking hydrophobic bonding is to provide extra stability and the ideal coordination required for optimal interaction of the DNP-poly(A) substrate with the catalytic residues of PARN.

In order to confirm the above findings the Polymer Property Predictor Tool (PPPT) of MOE suite was used [44]. The properties predicted by PPPT use the chemical and structural information per monomer repeat unit to simulate a polymer in an extended conformation. Connectivity indices alongside with structural fragment descriptors are used to predict the properties of monomer repeat unit, which are virtually connected as one polymer molecule.

It was determined that for the same molecular repeat unit of each nucleoside, the DNP-poly(A) has larger Van der Waals volume, higher steric hindrance parameter and higher molar stiffness (Fig. S4C and Table S4). However, since the DNP moiety is expected to be incorporated in one every five nucleosides [43], it was decided that for the purposes of the molecular dynamics simulations only the adenosine nucleotide that fits our pharmacophore model, would be converted to DNP-(A) in the catalytic site of PARN. The MDs equilibrium energy for the PARN-substrate complex, was found to be three times higher for DNP-poly(A), compared to the corresponding equilibrium energy for the natural substrate, the poly(A). All of the above explain the reduced activity observed for DNP-poly(A) when compared to poly(A).

### DNP-poly(A) is a Competitive Inhibitor of PARN

**Figure 5:**
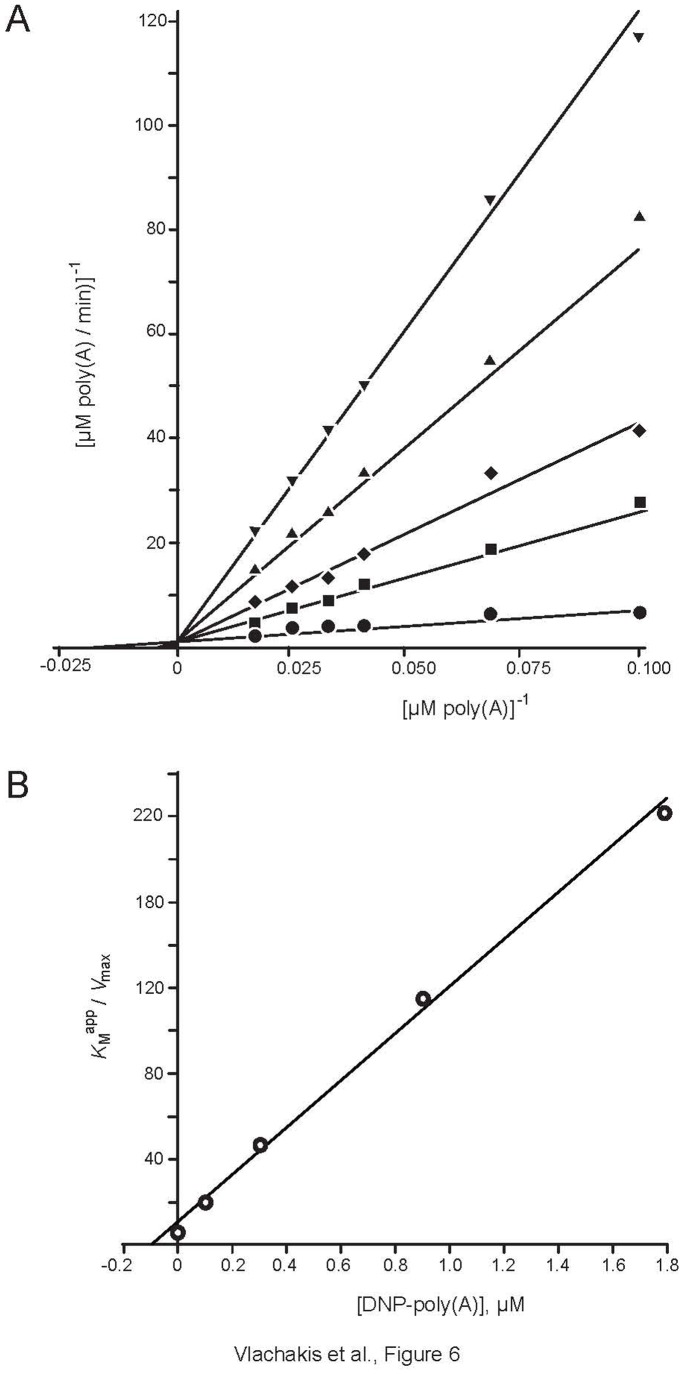
DNP-poly(A) is a competitive inhibitor of PARN. (A). Double reciprocal plots 1/v versus 1/[substrate] for PARN activity in the presence or absence (•) of DNP-poly(A) are shown. The DNP-poly(A) concentrations are 0.1 (▪), 0.3 (⧫) and 0.9 (▴) and 1.8 (▾) mM. Representative plots of at least three independent experiments. Substrate concentrations range from 0.1–0.6 mM poly(A). (B). The slopes (*K*_M_^app^/*V*_max_) of the double reciprocal lines are plotted versus the DNP-poly(A) concentration used to calculate the *K*_i_ value. The intercept of line on *x*-axis represents –*K*_i_.

To evaluate our prediction of the inhibitory properties of DNP-poly(A), we performed biochemical assays of PARN activity. Detailed kinetic analysis of the assays revealed that DNP-poly(A) behaves as a competitive inhibitor of PARN (Fig. 5). The calculated *K*_i_ value is 98±5 µM, which is an approximately three-fold increase when compared to poly(A), whose *K*_M_ value is ∼30 µM and in total proportion with the corresponding predicted MD equilibrium energies (PARN/poly(A): −10500 Kcal/mole and PARN/DNPpoly(A): −3000 Kcal/mole, Fig. S4D). Our data show that the predicted DNP-poly(A) can efficiently suppress PARN activity. Taken together with our previous reports, DNP-poly(A) reveals *K*_i_ value significantly improved when compared with some of the most efficient PARN inhibitors (Table S5). In fact, it is the second best inhibitor, after the slow-binding U1 competitive inhibitor. Importantly, the kinetic analysis supports the prediction of our pharmacophore that DNP-poly(A) may efficiently inhibit PARN, thus suggesting that it may be used for effective specific inhibitors with therapeutic potential, taking also into account the improved characteristics of the compound, such as cell permeability, and nuclease resistance.

### Conclusions

We developed a 3D pharmacophore model for PARN, due to its emerging potential as a biomarker and a pharmaceutical target suitable for drug design. We performed an in-depth phylogenetic and structural analysis of the catalytic mechanism of human PARN that rationalizes the available *in silico* and biochemical data. The pharmacophore predicted DNP-poly(A) as such a candidate and the kinetic analysis verified that the compound behaves as an efficient competitive inhibitor of PARN. The present work opens the field for the design of novel compounds with improved biochemical and clinical characteristics in the future.

## Methods

### Coordinate Preparation

3D coordinates were obtained from the X-ray solved, crystal structures of PARN with RCSB codes: 2A1S and 2A1R. The 2A1S entry is the full length, unbound form of PARN, whereas the 2A1R entry contains the catalytic domain of PARN, in bound form with a 3-mer poly(A) chain. The resolution of both X-ray structures is 2.6 Å overall. All the important parts of both structures, including the catalytic site and the underlying layer, are very clear in their electron densities. For the purposes of this study, the dimeric form of PARN was used in all calculations.

### Energy Minimization

Energy minimizations were used to remove any residual geometrical strain in each molecular system, using the Charmm27 forcefield as it is implemented into the Gromacs suite, version 4.5.5 [45]–[48]. All Gromacs-related simulations were performed though our previously developed graphical interface [49]. An implicit Generalized Born (GB) solvation was chosen at this stage, in an attempt to speed up the energy minimization process.

### Molecular Dynamics Simulations

Molecular systems were subjected to unrestrained Molecular Dynamics simulations (MDs) using the Gromacs suite, version 4.5.5 [45]–[48]. MDS took place in a SPC water-solvated, periodic environment. Water molecules were added using the truncated octahedron box extending 7 Å from each atom. Molecular systems were neutralized with counter-ions as required. For the purposes of this study all MDS were performed using the NVT ensemble in a canonical environment, at 300 K, 1 atm and a step size equal to 2 femtoseconds for a total 100 nanoseconds simulation time. An NVT ensemble requires that the Number of atoms, Volume and Temperature remain constant throughout the simulation.

### Sequence Database Search

A combination of key terms and BLAST searches were employed in order to identify homologous PARN protein sequences. The names and/or accession numbers of the characterized PARNs, including human [9], cattle [17], *Xenopus laevis* [50] and *Arabidopsis thaliana* [51] PARN, were used to retrieve their corresponding amino acid sequences from UniProtKB [52]. Subsequently, these sequences were used as probes to search the non-redundant databases UniProtKB [52] and GenBank [53] by applying reciprocal BLASTp and tBLASTn [54]. This process was reiterated until convergence.

### Phylogenetic Analysis

The retrieved PARN peptide sequences were searched against the InterPro database [55] to identify the boundaries of the catalytic nuclease domain. In order to optimize the sequence alignment, the predicted core nuclease domain was excised from the full-length protein and was used in our phylogenetic analysis. Subsequently, these trimmed sequences were aligned using CLUSTALW [56]. The resulting multiple sequence alignment was then submitted to ProtTest [57] in order to determine the optimal model for protein evolution. Then, a phylogenetic tree employing a maximum-likelihood method implemented in PhyML [58] was reconstructed using the LG amino acid substitution model [59] with four substitution rate categories; the gamma shape parameter (α) and the proportion of invariable sites were estimated from the data. Bootstrap analysis (500 pseudo-replicates) was performed to test the robustness of the inferred tree. The phylogenetic tree was visualized with Dendroscope [60].

### Motif Construction

Peptide sequences of the PARN family were aligned and edited by employing Utopia suite’s CINEMA alignment editor [61]. Sequence motifs were excised from this alignment and were submitted to Weblogo [62] in order to generate consensus sequences for these motifs.

### Calculation of Molecular Descriptors

A molecular database consisting of our previously reported inhibitors (Table S5) was designed. Then a conformational search was carried out on each one, using the CHARMM27 forcefield as implemented within the MOE package, in order to acquire the global energy minimum of each structure. Finally, the atomic contributions of a total of 330 molecular descriptors were calculated (for a full list of the descriptors used please refer to Table S2) using the implemented descriptor calculator module, as implemented in MOE suite [44].

### 2D Structure Activity Relationships and Statistical Analysis

Structure Activity Relationships (SAR) were calculated based on the coefficients of determination R^2^ and the Pearson’s contingency coefficients C between the Ki activity and the molecular electronic properties. R^2^ measures how well a regression line represents the data, whereas C measures the relative strength of association between two variables. The R^2^ values vary between 1 (strong linear association between the two variables) and 0 (weak linear association). The C values vary between 0 (uncorrelated) and 1 (strong correlation). In order to filter the descriptors with important contribution to the observed biological activity of each inhibitor, descriptors with R^2^>0.2 and C>0.6 were selected (coloured in red in the full list of descriptors presented in Table S3). Notably, most selected descriptors were quantifying the electronic, steric and hydrophobic properties of the 15 inhibitor compounds. These properties have been previously found to be important characteristics that explain the deadenylase activity of PARN and similar catalytic activities of relative enzymes [16], [26].

Data patterns between the different modules were then identified in the filtered, based on the above coefficients, data using hierarchical clustering. Additionally, Principal Components Analysis (PCA) was employed on descriptors with non-zero values. All statistical analysis for the estimation of SAR relationships has been conducted using the R statistical software [63].

### Hierarchical Clustering

Hierarchical clustering with resampling was applied to the filtered data to estimate clusters of compounds based on their correlations structures. The pvclust hierarchical clustering algorithm was employed as implemented within the R package [64]. For each cluster the algorithm calculates p-values via multiscale bootstrap resampling to test the robustness of the inferred clustering and report how strongly the cluster is supported by the data. By default pvclust performs hierarchical clustering K×B times, where K = 10 different data sizes and B = 1,000 denotes the number of bootstrap sample [64]. The algorithm provides two types of p-values, the Approximately Unbiased (AU) which are computed by multiscale bootstrap resampling and the Bootstrap Probability (BP) values which are computed by normal bootstrap resampling. Clusters with AU≥95% were selected, which are strongly supported by the data.

### Principal Component Analysis

Principal Components Analysis (PCA) was employed to identify a subspace that captures most of the variation in the data, and suppress information which is not presented [48], [65]. PCA is useful to distinguish between samples with multiple measurements. We performed PCA using the prcomp algorithm as implemented in R, to extract uncorrelated principal components by linear transformations of the original variables (descriptors) so that the first components account for a large proportion of the variability (80–90%) of the original data. The prcomp algorithm automatically centers the data. Correlation coefficients between the PC scores and the original variables measure the importance of each variable in accounting for the variability, whereas the loadings, or eigenvectors, indicate how variation in the measurements is aligned with variation in the PC axes.

### Drug Likeness Correlation

Drug likeness was calculated based on Lipinsky’ s rule of five [66]. Molecular weight, number of donor/acceptor atoms and the logP of each compound (Table S3) were estimated using MOE suite. Furthermore, the drug potential of our training set was tested by an assessment of the toxicity or mutagenicity of the ligand using a rule-based method [67] and an estimated ease of synthesis as a percentage of heavy atoms traced to starting materials after retrosynthetic analysis, as implemented in MOE. Compounds that were either predicted to be toxic or hard to synthesize were neglected from the SAR statistical correlation.

### Pharmacophore Elucidation

We used all, of our previously published, nucleoside-analog inhibitors, alongside the current 2D statistical analyses for the Pharmacophore design of PARN [16], [26]. The biological evaluation of those compounds produced quite diverse results, ranging from highly potent inhibitors (i.e. U1, *K*_i_ = 19) to rather inactive or even activating ones (i.e. A7, *K*_i_>1 mM). The atomic contributions calculated above (as molecular descriptors) were applied to the whole structure of each compound. The “Complexed-based” pharmacophore module of MOE suite was used in this study, incorporating the docking conformations of our compounds as previously described [16], [26]. Initially, a series of Pharmacophore Annotation Points (PAPs) were made for each compound. Then PAPs common among the most active compounds were retained, whereas PAPs in least active ones were discarded. The highest ranking 3D pharmacophore hypotheses, as a grouped 3D arrangement of PAPs was selected, since it presented the best correlation to the pharmacological activities of our inhibitor compounds.

### Homology Modelling

The homology modelling of the *Arabidopsis thaliana* and *Trypanosoma brucei* PARN enzymes was carried out using Modeller [68]. The crystal structure of the human PARN was used as template (RCSB entry: 2A1R). Subsequent energy minimization was performed using the Gromacs-implemented, Charmm27 forcefield. Models were structurally evaluated using the Procheck ulitily [69].

### Synthesis of Poly[2′-O-(2,4-dinitrophenyl)]poly-(A), DNP-poly(A)

DNP-poly(A) was synthesized as previously described [43]. In brief, the synthesis was based on poly(A) (supplied by Sigma; average size 300 adenosines, A_300_, equal to a molecular weight 10^5^). The average molecular weight of DNP-poly(A) was estimated to be 1.1⋅10^5^ according to the previously determined DNP-to-adenine ratio [43]. The difference in the molecular weights A_300_ and DNP-poly(A) indicates that approximately 60 out of 300 adenosines bear a DNP moiety, thus 1 every 5 adenosines is converted to DNP-adenosine.

### PARN Activity Assay and Kinetic Analysis

The enzymatic activity was determined by the spectrophotometric methylene blue assay as described before [70]. Deadenylation rates as a function of time were determined with time-course assays. The reactions were performed using 0.01–0.02 mM recombinant PARN and the substrate concentration [poly (A)] varied from 0.1 to 0.6 mM [16]. DNP-poly(A) concentrations varied from 0.082 to 3 mΜ.

## Supporting Information

Figure S1 **The homology models of** ***Arabidopsis thaliana*** **and** ***Trypanosoma brucei*** **PARN monomers in ribbon representation, superposed on the human PARN (RCSB entry: 2A1R).** The human PARN is colored orange, the *Arabidopsis thaliana* PARN is in cream color and the *Trypanosoma brucei* PARN monomer is colored blue. R99 of human PARN and R89 of the *Arabidopsis thaliana* PARN share the same spatial coordinates, which confirms the structural conservation of that amino acid in the *Arabidopsis thaliana* PARN too.
(TIF)

Figure S2 **Ligplot interaction maps of the four oligonucleotides: poly(A), poly(U), poly(C) and poly(G) in the same catalytic site of human PARN.** Only the PARN-poly(A) complex managed to incorporate the crystallographic waters that could be occupying the site where divalent M^2+^ metal ions are expected to bind, as well as establish H-bonding interactions with the Arg99 residue.
(TIF)

Figure S3 **Identification of correlation structures and measures variability among the 15 compounds examined.** (A) Hierarchical clustering of the compounds based on the pairwise correlations of the filtered data. Values on the edges of the clustering are AU (red) and BP (green) p-values. Clusters with AU≥95% are indicated by rectangles. (B) PCA loading plots showing the data relative to the first three PCs. In accordance with A, the members of the non-adenosine inhibitors are forming a single group in both instances. (C) Density plots of Ki activity, Molecular Weight and LogP with respect to the adenosine inhibitors. The plot demonstrates evident association relationships between the three measures.
(TIF)

Figure S4 **DNP-poly(A) polymer as a novel anti-PARN agent.** (A) The poly(A) and DNP-poly(A) monomers. The four atoms participating in the dihedral energy plots are highlighted with arrows. (B) Dihedral angle plots for poly(A) and DNP-poly(A) *in vacuo* and the active site of PARN (C) Normalized polymer comparison between poly(A) and DNP-poly(A). (D) Molecular dynamics simulation of the PARN - poly(A) and PARN - DNP-poly(A) complexes.
(TIF)

Figure S5 **The arrangement of the first scissile bond and the first nucleotide of the poly(A) substrate in the catalytic site of PARN.** (A) The poly(A) substrate is fixed with hydrogen bonding interactions with the Arg99 and His377 amino acids. Phe31 residue is in close proximity but doesn’t interact with the poly(A) substrate. (B) The DNP-poly(A) substrate interacts with the Arg99 and His377 amino acids by hydrogen bonding and the Phe31 residue by pi-stacking hydrophobic interactions.
(TIF)

Table S1 **Phylogenetic distribution of the PARN proteins analyzed in the present study.** The *Drosophila melanogaster* and *Saccharomyces cerevisiae* POP2 sequences are shown in green.
(DOCX)

Table S2 **List of the 330 molecular descriptors and the calculated values that were used in our statistical analysis.** Indicated with red colour are the inhibitors most highly correlated with Ki based on R^2^ and C coefficients.
(DOCX)

Table S3 **Drug likeness properties of our previously reported nucleoside analog inhibitors of PARN, including the consensus score of drug likeness, a toxicity measure and an ease-of-synthesis approximation.**
(DOCX)

Table S4 **Poly(A) and DNP-poly(A) polymer properties prediction values.**
(DOCX)

Table S5 **Summary of the compounds and their corresponding inhibition constants used in our statistical analysis and pharmacophore design.**
(DOCX)

Table S6 **Summary of the inhibitor compounds of table S5 and their corresponding interaction energies with the catalytic site of PARN.** Interaction energies (*Int*. *E*.) are represented in Kcal/mole units and have been calculated using the potential energy module of MOE.
(DOCX)

Text S1 **Statistical evaluation of structure activity relationships.**
(DOCX)
