# Supplementary figures and images for: An Integrated In Silico Approach to Design Specific Inhibitors Targeting Human Poly(A)-Specific Ribonuclease
Source: PLoS One. 2012 Dec 6;7(12):e51113. doi: 10.1371/journal.pone.0051113 (PMC3516499; doi:10.1371/journal.pone.0051113)

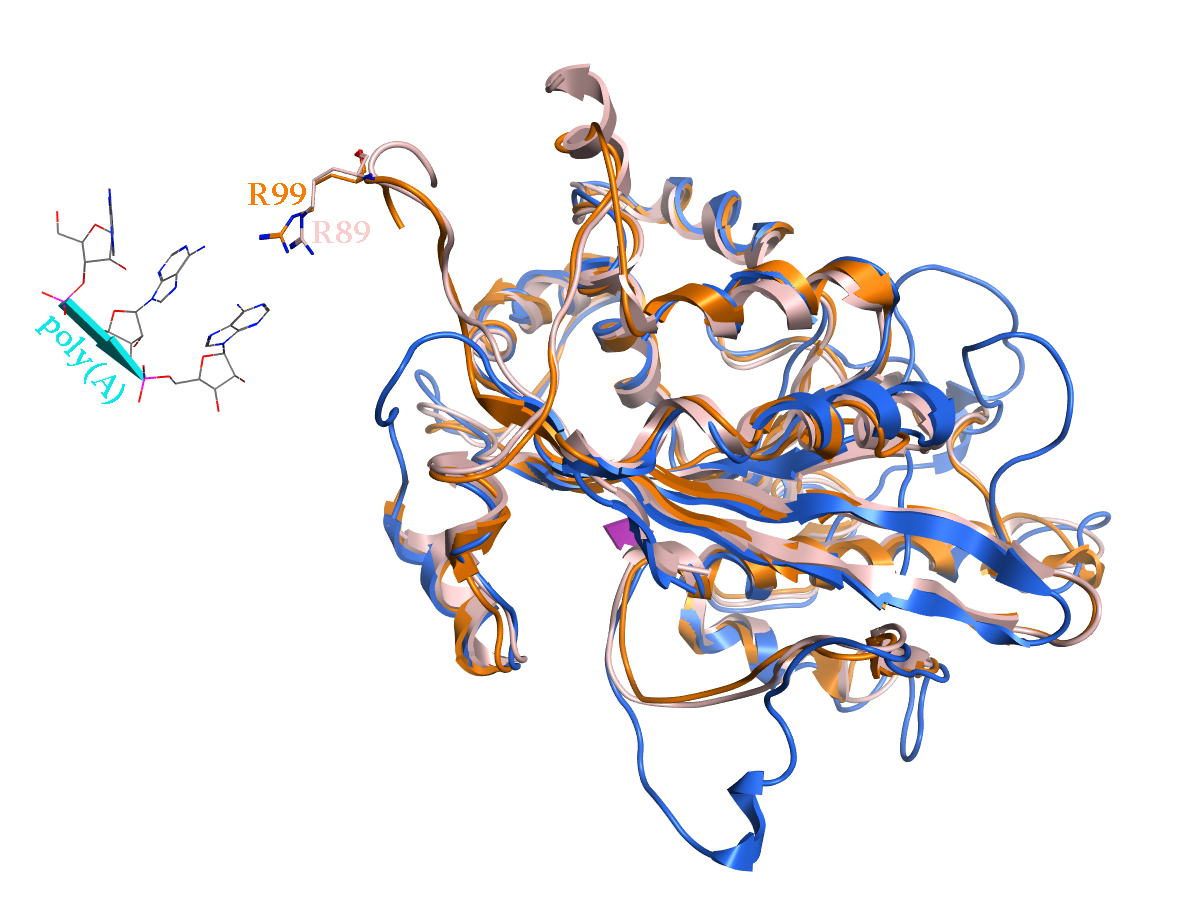

Supplement: Figure S1 — The homology models of Arabidopsis thaliana and Trypanosoma brucei PARN monomers in ribbon representation, superposed on the human PARN (RCSB entry: 2A1R). The human PARN is colored orange, the Arabidopsis thaliana PARN is in cream color and the Trypanosoma brucei PARN monomer is colored blue. R99 of human PARN and R89 of the Arabidopsis thaliana PARN share the same spatial coordinates, which confirms the structural conservation of that amino acid in the Arabidopsis thaliana PARN too. (TIF) [file pone.0051113.s001.tif]

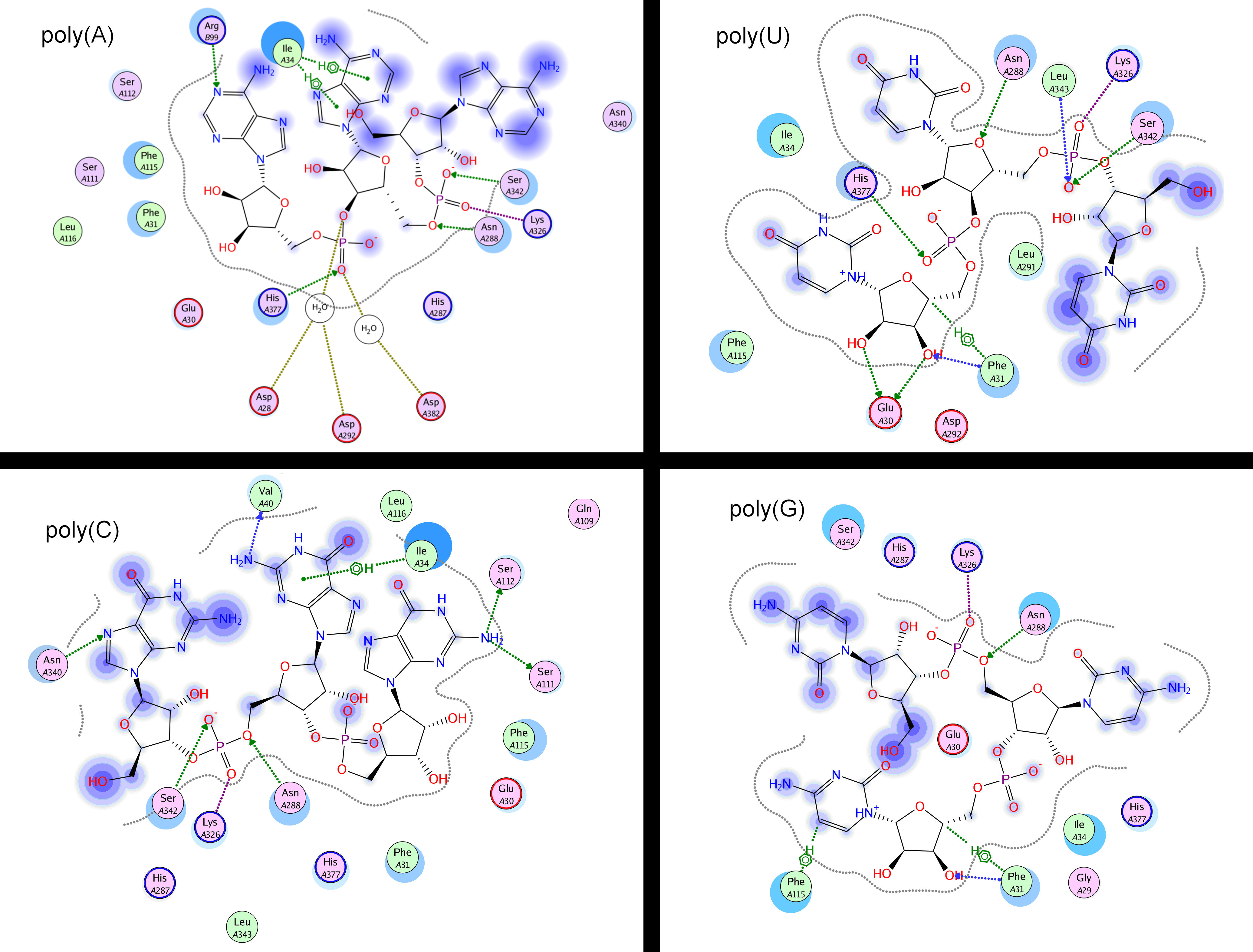

Supplement: Figure S2 — Ligplot interaction maps of the four oligonucleotides: poly(A), poly(U), poly(C) and poly(G) in the same catalytic site of human PARN. Only the PARN-poly(A) complex managed to incorporate the crystallographic waters that could be occupying the site where divalent M2+ metal ions are expected to bind, as well as establish H-bonding interactions with the Arg99 residue. (TIF) [file pone.0051113.s002.tif]

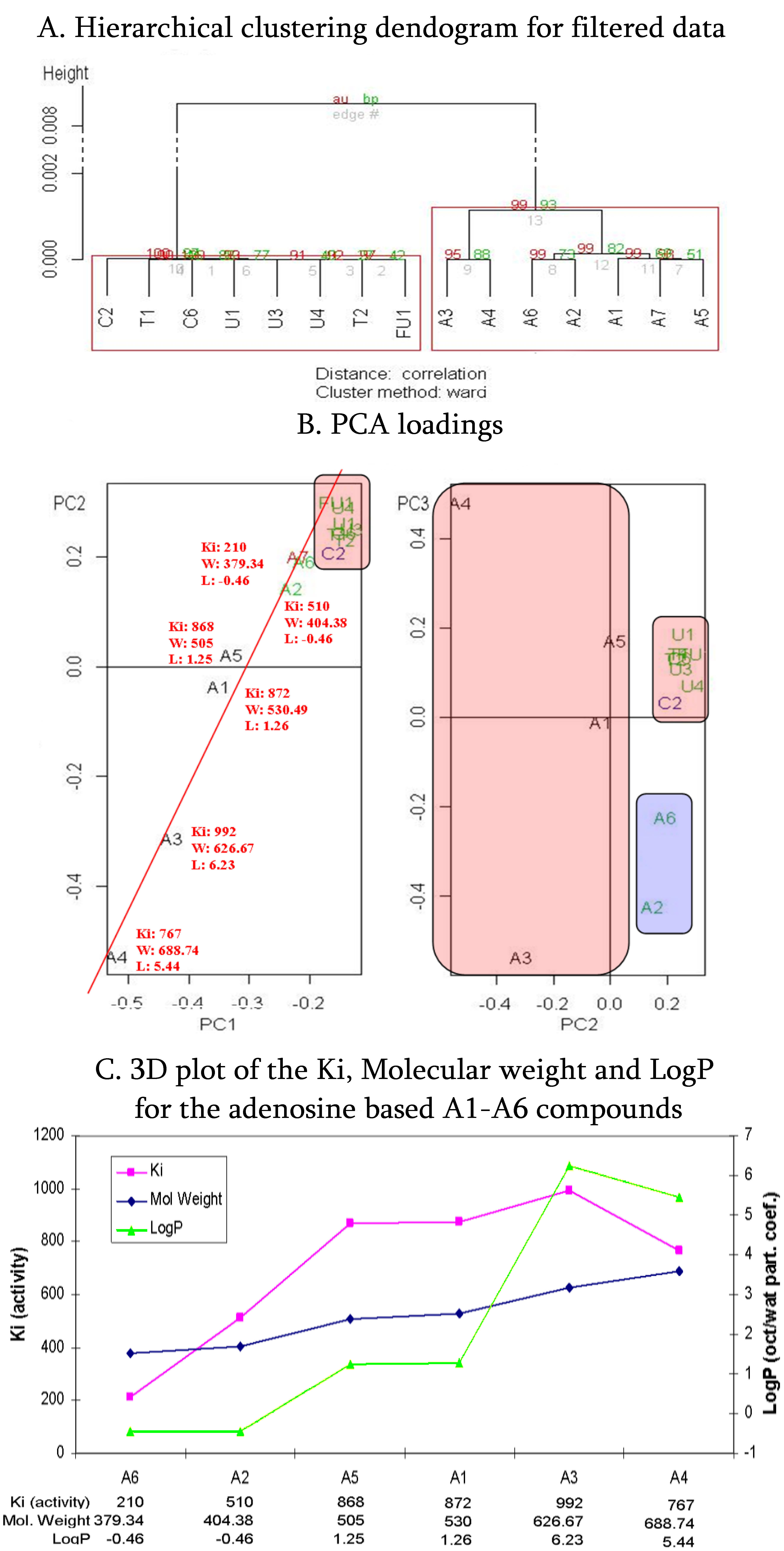

Supplement: Figure S3 — Identification of correlation structures and measures variability among the 15 compounds examined. (A) Hierarchical clustering of the compounds based on the pairwise correlations of the filtered data. Values on the edges of the clustering are AU (red) and BP (green) p-values. Clusters with AU≥95% are indicated by rectangles. (B) PCA loading plots showing the data relative to the first three PCs. In accordance with A, the members of the non-adenosine inhibitors are forming a single group in both instances. (C) Density plots of Ki activity, Molecular Weight and LogP with respect to the adenosine inhibitors. The plot demonstrates evident association relationships between the three measures. (TIF) [file pone.0051113.s003.tif]

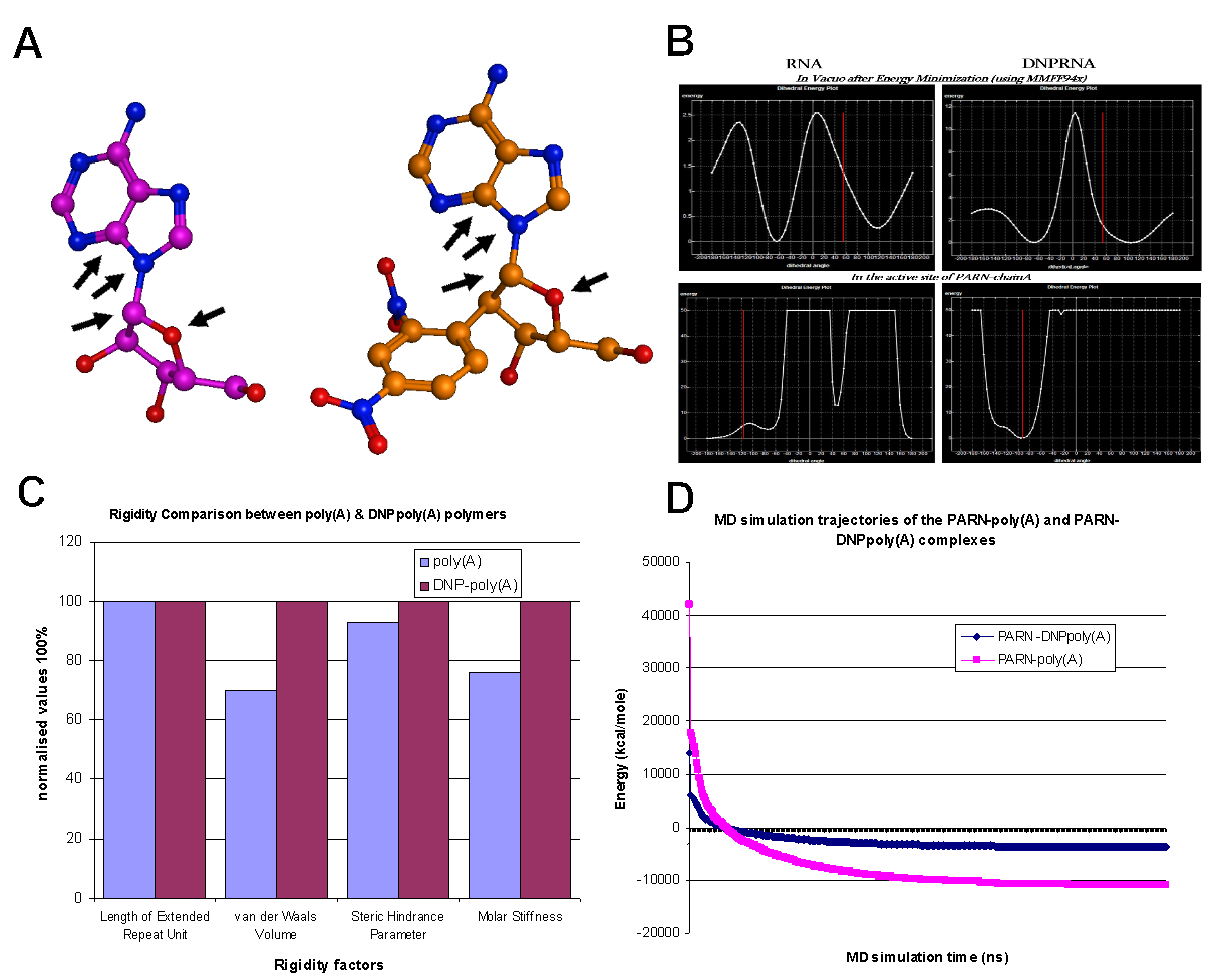

Supplement: Figure S4 — DNP-poly(A) polymer as a novel anti-PARN agent. (A) The poly(A) and DNP-poly(A) monomers. The four atoms participating in the dihedral energy plots are highlighted with arrows. (B) Dihedral angle plots for poly(A) and DNP-poly(A) in vacuo and the active site of PARN (C) Normalized polymer comparison between poly(A) and DNP-poly(A). (D) Molecular dynamics simulation of the PARN - poly(A) and PARN - DNP-poly(A) complexes. (TIF) [file pone.0051113.s004.tif]

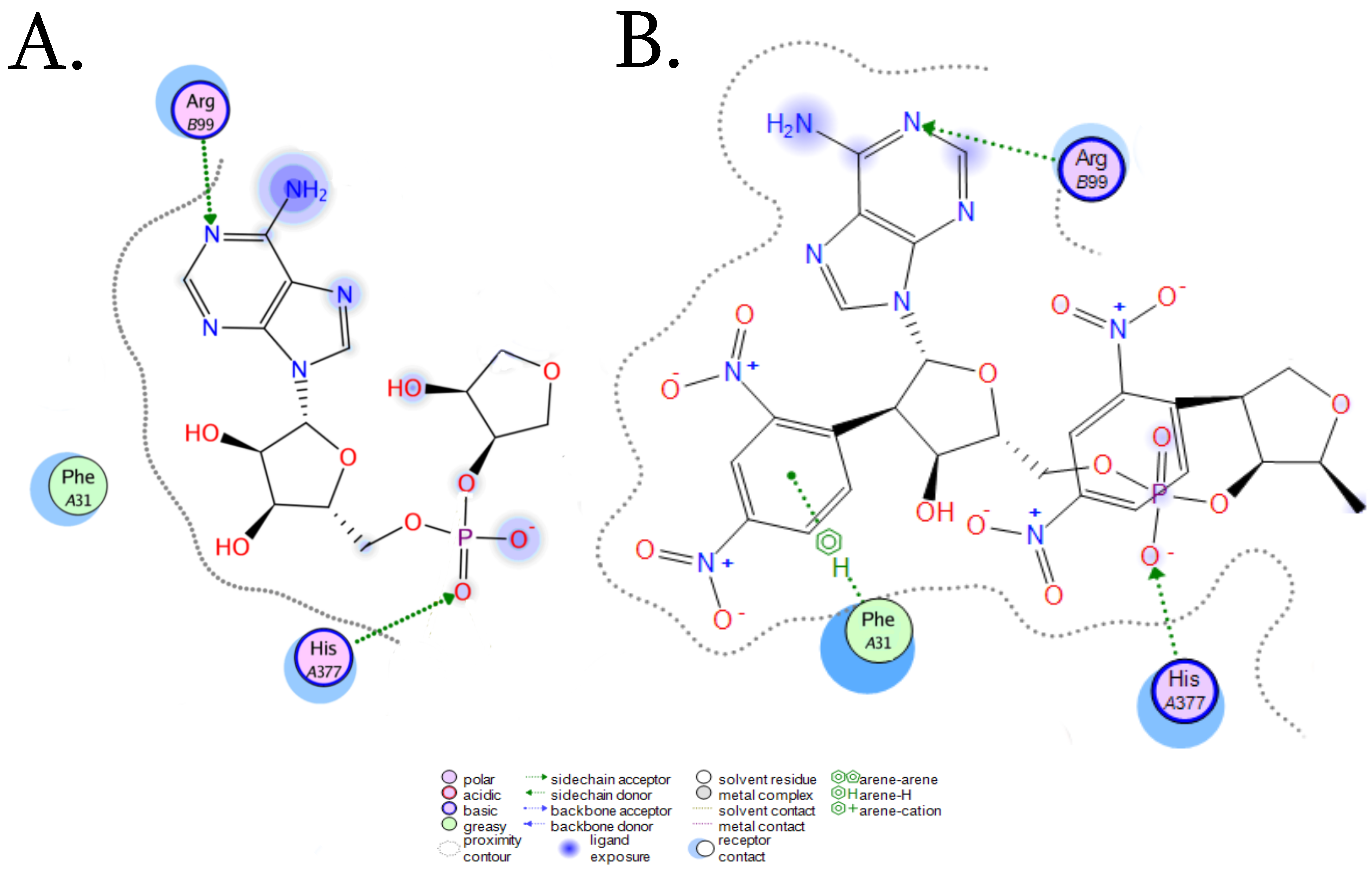

Supplement: Figure S5 — The arrangement of the first scissile bond and the first nucleotide of the poly(A) substrate in the catalytic site of PARN. (A) The poly(A) substrate is fixed with hydrogen bonding interactions with the Arg99 and His377 amino acids. Phe31 residue is in close proximity but doesn’t interact with the poly(A) substrate. (B) The DNP-poly(A) substrate interacts with the Arg99 and His377 amino acids by hydrogen bonding and the Phe31 residue by pi-stacking hydrophobic interactions. (TIF) [file pone.0051113.s005.tif]
