# Supplementary material for: An Integrated In Silico Approach to Design Specific Inhibitors Targeting Human Poly(A)-Specific Ribonuclease
Source: PLoS One. 2012 Dec 6;7(12):e51113. doi: 10.1371/journal.pone.0051113 (PMC3516499; doi:10.1371/journal.pone.0051113)
Supplement: Table S1 — Phylogenetic distribution of the PARN proteins analyzed in the present study. The Drosophila melanogaster and Saccharomyces cerevisiae POP2 sequences are shown in green. (DOCX) [file pone.0051113.s006.docx]

**Table S1**

| **Taxonomy/Species** | **Accession number** |
| --- | --- |
| **1. Metazoa** |  |
| **1.1. Chordata** |  |
| **1.1.1. Mammalia** |  |
| **1.1.1.1. Eutheria** |  |
| *Homo sapiens* (Human) | O95453^1^ |
| *Mus musculus* (Mouse) | Q8VDG3^1^ |
| *Bos taurus* (Cattle) | P69341^1^ |
| *Canis lupus familiaris* (Dog) | E2QZA0^1^ |
| *Loxodonta africana* (Elephant) | G3TEC9^1^ |
| *Myotis lucifugus* (Bat) | G1P4Q5^1^ |
| **1.1.1.2. Metatheria** |  |
| *Monodelphis domestica* (Opossum) | F7BH34^1^ |
| **1.1.1.3. Prototheria** |  |
| *Ornithorhynchus anatinus* (Platypus) | F6UWG5^1^ |
| **1.1.2. Sauria** |  |
| **1.1.2.1. Aves** |  |
| *Gallus gallus* (Chicken) | F1NEI8^1^ |
| **1.1.2.2. Lepidosauria** |  |
| *Anolis carolinensis* (Lizard) | XP_003228705.1 ^2^ |
| **1.1.3. Amphibia** |  |
| *Xenopus laevis* (Frog) | Q90ZA1^1^ |
| **1.1.4. Actinopterygii** |  |
| *Danio rerio* (Zebrafish) | Q7ZU92^1^ |
| **1.1.5. Tunicata** |  |
| *Ciona intestinalis* (Sea squirt) | F6PI67^1^ |
| **1.2. Nematoda** |  |
| *Caenorhabditis elegans* (Nematode) | Q21412^1^ |
| **1.3. Echinodermata** |  |
| *Strongylocentrotus purpuratus* (Sea urchin) | XP_785661.2 ^2^ |
| **1.4. Cnidaria** |  |
| *Nematostella vectensis* (Sea anemone) | A7T0T2^1^ |
| **1.5. Arthropoda** |  |
| *Apis mellifera* (Honeybee) | XP_392167.3 ^2^ |
| *Anopheles gambiae* (Mosquito) | Q7PT32^1^ |
| ***Drosophila melanogaster* (Fruit fly)** | **Q9VTS4^1^** |
| **2. Fungi** |  |
| ***Saccharomyces cerevisiae* (Yeast)** | **P39008^1^** |
| *Schizosaccharomyces pombe* (Fission yeast) | O94386^1^ |
| *Aspergillus niger* | G3YAF6^1^ |
| *Neurospora crassa* | Q7S1Z4^1^ |
| **3. Viridiplantae** |  |
| **3.1. Tracheophyta** |  |
| **3.1.1. Eudicotyledons** |  |
| *Arabidopsis thaliana* | Q9LG26^1^ |
| **3.1.2. Moncotyledons** |  |
| *Oryza sativa* (Rice) | Q01KE8^1^ |
| *Zea mays* (Maize) | C0PFF8^1^ |
| **3.2. Bryophyta** |  |
| *Physcomitrella patens* (Moss) | A9SGS0^1^ |
| **3.3. Chlorophyta** |  |
| *Volvox carteri* (Green alga) | D8TSI7^1^ |
| **4. Stramenopiles** |  |
| **4.1. Phaeophyceae** |  |
| *Ectocarpus siliculosus* (Brown alga) | D7FNN0^1^ |
| **4.2. Oomycetes** |  |
| *Phytophthora sojae* | **G4Z0L6**^1^ |
| **5. Euglenozoa** |  |
| *Trypanosoma brucei* | Q38AC8^1^ |
| *Leishmania major* | Q4Q0C3^1^ |
| **6. Alveolata** |  |
| *Theileria parva* | Q4N4G4^1^ |
| **7. Heterolobosea** |  |
| *Naegleria gruberi* (Amoeba) | D2V2U5^1^ |

^1^UniProtKB: <http://www.uniprot.org/>; ^2^NCBI: <http://www.ncbi.nlm.nih.gov/>
