# Supplementary material for: An Integrated In Silico Approach to Design Specific Inhibitors Targeting Human Poly(A)-Specific Ribonuclease
Source: PLoS One. 2012 Dec 6;7(12):e51113. doi: 10.1371/journal.pone.0051113 (PMC3516499; doi:10.1371/journal.pone.0051113)
Supplement: Table S2 — List of the 330 molecular descriptors and the calculated values that were used in our statistical analysis. Indicated with red colour are the inhibitors most highly correlated with Ki based on R2 and C coefficients. (DOCX) [file pone.0051113.s007.docx]

**Table S2**

|  | A3 | A7 | T2 | T1 | U4 | FU1 | C6 | A6 | U3 | U1 | C2 | A2 | A4 | A5 | A1 |
| --- | --- | --- | --- | --- | --- | --- | --- | --- | --- | --- | --- | --- | --- | --- | --- |
| Ki | 992.43 | 1111.89 | 1000.00 | 135.00 | 1000.00 | 98.00 | 645.00 | 210.00 | 1000.00 | 19.00 | 1000.00 | 510.00 | 767.30 | 868.00 | 872.97 |
| MM | 14.90 | 3.82 | 5.25 | 5.58 | 5.23 | 5.37 | 6.75 | 3.86 | 5.36 | 5.66 | 9.82 | 3.60 | 11.76 | 7.61 | 8.58 |
| a_acc | 0.00 | 0.00 | 0.00 | 0.00 | 0.00 | 0.00 | 0.00 | 0.00 | 0.00 | 0.00 | 0.00 | 0.00 | 0.00 | 0.00 | 0.00 |
| a_acid | 33.00 | 6.00 | 0.00 | 0.00 | 0.00 | 0.00 | 0.00 | 6.00 | 0.00 | 0.00 | 9.00 | 15.00 | 33.00 | 6.00 | 15.00 |
| a_aro | 3.00 | 0.00 | 0.00 | 0.00 | 0.00 | 0.00 | 0.00 | 0.00 | 0.00 | 0.00 | 3.00 | 3.00 | 3.00 | 0.00 | 3.00 |
| a_base | 76.00 | 45.00 | 34.00 | 35.00 | 31.00 | 32.00 | 33.00 | 45.00 | 31.00 | 32.00 | 36.00 | 48.00 | 86.00 | 60.00 | 63.00 |
| a_count | 0.00 | 1.00 | 3.00 | 4.00 | 3.00 | 4.00 | 4.00 | 4.00 | 3.00 | 4.00 | 3.00 | 3.00 | 1.00 | 1.00 | 0.00 |
| a_don | 47.00 | 29.00 | 19.00 | 20.00 | 19.00 | 20.00 | 19.00 | 27.00 | 18.00 | 19.00 | 21.00 | 29.00 | 51.00 | 36.00 | 38.00 |
| a_heavy | 31.00 | 14.00 | 9.00 | 9.00 | 9.00 | 9.00 | 8.00 | 14.00 | 8.00 | 8.00 | 7.00 | 13.00 | 33.00 | 17.00 | 16.00 |
| a_hyd | 121.60 | 82.16 | 62.71 | 65.36 | 61.73 | 64.25 | 63.58 | 82.32 | 58.65 | 61.17 | 69.85 | 89.09 | 139.88 | 107.05 | 114.82 |
| a_IC | 1.60 | 1.83 | 1.84 | 1.87 | 1.99 | 2.01 | 1.93 | 1.83 | 1.89 | 1.91 | 1.94 | 1.86 | 1.63 | 1.78 | 1.82 |
| a_ICM | 0.00 | 0.00 | 0.00 | 0.00 | 0.00 | 0.00 | 0.00 | 0.00 | 0.00 | 0.00 | 0.00 | 0.00 | 0.00 | 0.00 | 0.00 |
| a_nB | 0.00 | 0.00 | 0.00 | 0.00 | 0.00 | 0.00 | 0.00 | 0.00 | 0.00 | 0.00 | 0.00 | 0.00 | 0.00 | 0.00 | 0.00 |
| a_nBr | 37.00 | 19.00 | 11.00 | 11.00 | 10.00 | 10.00 | 10.00 | 17.00 | 10.00 | 10.00 | 11.00 | 18.00 | 39.00 | 23.00 | 24.00 |
| a_nC | 0.00 | 0.00 | 0.00 | 0.00 | 0.00 | 0.00 | 0.00 | 0.00 | 0.00 | 0.00 | 0.00 | 0.00 | 0.00 | 0.00 | 0.00 |
| a_nCl | 1.00 | 1.00 | 1.00 | 1.00 | 2.00 | 2.00 | 1.00 | 1.00 | 1.00 | 1.00 | 1.00 | 1.00 | 1.00 | 1.00 | 1.00 |
| a_nF | 29.00 | 16.00 | 15.00 | 15.00 | 12.00 | 12.00 | 14.00 | 18.00 | 13.00 | 13.00 | 15.00 | 19.00 | 35.00 | 24.00 | 25.00 |
| a_nH | 0.00 | 0.00 | 0.00 | 0.00 | 0.00 | 0.00 | 0.00 | 0.00 | 0.00 | 0.00 | 0.00 | 0.00 | 0.00 | 0.00 | 0.00 |
| a_nI | 5.00 | 3.00 | 2.00 | 2.00 | 2.00 | 2.00 | 3.00 | 3.00 | 2.00 | 2.00 | 5.00 | 5.00 | 5.00 | 3.00 | 5.00 |
| a_nN | 4.00 | 6.00 | 5.00 | 6.00 | 5.00 | 6.00 | 5.00 | 6.00 | 5.00 | 6.00 | 4.00 | 5.00 | 6.00 | 9.00 | 8.00 |
| a_nO | 0.00 | 0.00 | 0.00 | 0.00 | 0.00 | 0.00 | 0.00 | 0.00 | 0.00 | 0.00 | 0.00 | 0.00 | 0.00 | 0.00 | 0.00 |
| a_nP | 0.00 | 0.00 | 0.00 | 0.00 | 0.00 | 0.00 | 0.00 | 0.00 | 0.00 | 0.00 | 0.00 | 0.00 | 0.00 | 0.00 | 0.00 |
| a_nS | 1.05 | 1.52 | 2.12 | 2.14 | 2.12 | 2.14 | 2.10 | 1.55 | 2.08 | 2.10 | 1.77 | 1.41 | 1.11 | 1.70 | 1.50 |
| AM1_dipole | -182763.38 | -130953.52 | -94935.68 | -102331.31 | -102207.59 | -109603.05 | -96427.88 | -125731.65 | -91341.85 | -98737.34 | -101689.66 | -130992.52 | -205407.45 | -167515.98 | -172773.22 |
| AM1_E | -1776640.60 | -902589.25 | -578644.13 | -630869.06 | -586830.44 | -638987.44 | -579251.44 | -867032.25 | -532372.25 | -583186.00 | -651630.63 | -949088.19 | -2158066.80 | -1359250.30 | -1453876.40 |
| AM1_Eele | 217.22 | -155.58 | -225.69 | -284.88 | -271.06 | -317.52 | -221.69 | -218.02 | -231.21 | -277.71 | 0.70 | 5.41 | 100.71 | -324.05 | -96.11 |
| AM1_HF | -10.93 | -9.47 | -9.46 | -9.55 | -9.64 | -9.73 | -9.39 | -9.49 | -9.81 | -9.89 | -13.11 | -12.29 | -10.90 | -9.40 | -12.28 |
| AM1_HOMO | 10.93 | 9.47 | 9.46 | 9.55 | 9.64 | 9.73 | 9.39 | 9.49 | 9.81 | 9.89 | 13.11 | 12.29 | 10.90 | 9.40 | 12.28 |
| AM1_IP | -5.01 | -0.71 | -0.16 | -0.23 | -0.50 | -0.58 | -0.18 | -0.74 | -0.19 | -0.27 | -4.81 | -4.82 | -4.66 | -0.66 | -4.89 |
| AM1_LUMO | 93.72 | 52.78 | 36.13 | 36.93 | 32.93 | 33.73 | 34.80 | 50.59 | 33.04 | 33.84 | 38.63 | 54.42 | 102.85 | 67.56 | 71.38 |
| apol | 956.19 | 656.17 | 460.34 | 472.10 | 438.53 | 444.27 | 447.80 | 604.43 | 433.03 | 438.03 | 471.60 | 632.42 | 1019.33 | 779.24 | 807.18 |
| ASA | 541.39 | 254.92 | 139.75 | 127.55 | 151.35 | 155.04 | 189.61 | 195.85 | 158.40 | 163.86 | 252.05 | 326.21 | 513.24 | 266.66 | 339.28 |
| ASA- | 776.01 | 446.60 | 199.18 | 228.38 | 170.60 | 169.89 | 160.15 | 379.28 | 163.62 | 162.23 | 181.26 | 365.67 | 843.30 | 540.43 | 545.66 |
| ASA_H | 180.18 | 209.57 | 261.16 | 243.71 | 267.93 | 274.38 | 287.65 | 225.15 | 269.42 | 275.80 | 290.34 | 266.75 | 176.03 | 238.81 | 261.52 |
| ASA_P | 6.00 | 6.00 | 5.00 | 6.00 | 5.00 | 6.00 | 6.00 | 7.00 | 5.00 | 6.00 | 6.00 | 7.00 | 7.00 | 7.00 | 7.00 |
| ASA+ | 392.52 | 288.27 | 166.94 | 161.69 | 186.87 | 189.45 | 158.72 | 275.11 | 174.33 | 175.66 | 110.39 | 201.49 | 372.88 | 279.12 | 241.01 |
| b_1rotN | 0.17 | 0.13 | 0.05 | 0.10 | 0.05 | 0.10 | 0.10 | 0.10 | 0.05 | 0.10 | 0.09 | 0.13 | 0.18 | 0.16 | 0.17 |
| b_1rotR | 34.00 | 6.00 | 0.00 | 0.00 | 0.00 | 0.00 | 0.00 | 6.00 | 0.00 | 0.00 | 10.00 | 16.00 | 34.00 | 6.00 | 16.00 |
| b_ar | 82.00 | 47.00 | 35.00 | 36.00 | 32.00 | 33.00 | 34.00 | 47.00 | 32.00 | 33.00 | 38.00 | 51.00 | 92.00 | 62.00 | 66.00 |
| b_count | 3.00 | 7.00 | 3.00 | 3.00 | 3.00 | 3.00 | 3.00 | 4.00 | 3.00 | 3.00 | 0.00 | 1.00 | 2.00 | 7.00 | 4.00 |
| b_double | 53.00 | 31.00 | 20.00 | 21.00 | 20.00 | 21.00 | 20.00 | 29.00 | 19.00 | 20.00 | 23.00 | 32.00 | 57.00 | 38.00 | 41.00 |
| b_heavy | 10.00 | 7.00 | 1.00 | 2.00 | 1.00 | 2.00 | 2.00 | 5.00 | 1.00 | 2.00 | 2.00 | 5.00 | 12.00 | 11.00 | 11.00 |
| b_rotN | 0.19 | 0.23 | 0.05 | 0.10 | 0.05 | 0.10 | 0.10 | 0.17 | 0.05 | 0.10 | 0.09 | 0.16 | 0.21 | 0.29 | 0.27 |
| b_rotR | 45.00 | 34.00 | 32.00 | 33.00 | 29.00 | 30.00 | 31.00 | 37.00 | 29.00 | 30.00 | 28.00 | 34.00 | 56.00 | 49.00 | 46.00 |
| b_single | 0.00 | 0.00 | 0.00 | 0.00 | 0.00 | 0.00 | 0.00 | 0.00 | 0.00 | 0.00 | 0.00 | 0.00 | 0.00 | 0.00 | 0.00 |
| b_triple | 4865.48 | 1594.56 | 674.99 | 616.06 | 731.01 | 810.85 | 1074.54 | 1098.71 | 765.07 | 854.68 | 1550.60 | 2322.31 | 5075.42 | 2071.95 | 2741.06 |
| balabanJ | -2.54 | -2.53 | -2.71 | -2.71 | -2.71 | -2.71 | -2.71 | -2.71 | -2.71 | -2.71 | -2.67 | -2.68 | -2.68 | -2.71 | -2.68 |
| BCUT_PEOE_0 | -0.65 | -0.61 | -0.49 | -0.51 | -0.50 | -0.58 | -0.57 | -0.65 | -0.49 | -0.55 | -0.56 | -0.64 | -0.65 | -0.60 | -0.59 |
| BCUT_PEOE_1 | 0.70 | 0.66 | 0.57 | 0.60 | 0.59 | 0.62 | 0.66 | 0.70 | 0.60 | 0.63 | 0.68 | 0.71 | 0.70 | 0.65 | 0.66 |
| BCUT_PEOE_2 | 2.62 | 2.60 | 2.79 | 2.79 | 2.79 | 2.80 | 2.79 | 2.79 | 2.79 | 2.79 | 2.80 | 2.80 | 2.81 | 2.81 | 2.82 |
| BCUT_PEOE_3 | -2.63 | -2.68 | -2.89 | -2.91 | -2.89 | -2.91 | -2.90 | -2.90 | -2.89 | -2.91 | -2.85 | -2.85 | -2.85 | -2.90 | -2.85 |
| BCUT_SLOGP_0 | -0.54 | -0.54 | -0.51 | -0.54 | -0.51 | -0.54 | -0.65 | -0.57 | -0.51 | -0.54 | -0.65 | -0.58 | -0.54 | -0.56 | -0.56 |
| BCUT_SLOGP_1 | 0.81 | 0.70 | 0.48 | 0.55 | 0.48 | 0.55 | 0.53 | 0.65 | 0.48 | 0.55 | 0.53 | 0.65 | 0.77 | 0.62 | 0.62 |
| BCUT_SLOGP_2 | 2.61 | 2.46 | 2.61 | 2.59 | 2.61 | 2.59 | 2.59 | 2.59 | 2.61 | 2.59 | 2.64 | 2.64 | 2.67 | 2.61 | 2.66 |
| BCUT_SLOGP_3 | -2.31 | -2.30 | -2.51 | -2.51 | -2.51 | -2.51 | -2.51 | -2.51 | -2.51 | -2.51 | -2.50 | -2.50 | -2.51 | -2.51 | -2.50 |
| BCUT_SMR_0 | -0.50 | -0.45 | -0.37 | -0.38 | -0.38 | -0.41 | -0.46 | -0.48 | -0.38 | -0.41 | -0.46 | -0.49 | -0.50 | -0.45 | -0.45 |
| BCUT_SMR_1 | 0.85 | 0.79 | 0.64 | 0.66 | 0.66 | 0.73 | 0.73 | 0.85 | 0.66 | 0.73 | 0.76 | 0.85 | 0.85 | 0.78 | 0.79 |
| BCUT_SMR_2 | 2.83 | 2.82 | 2.97 | 2.97 | 2.97 | 2.97 | 2.97 | 2.97 | 2.97 | 2.97 | 2.96 | 2.96 | 2.97 | 2.99 | 2.98 |
| BCUT_SMR_3 | 44.59 | 30.32 | 24.07 | 24.07 | 22.00 | 22.00 | 22.02 | 28.67 | 21.89 | 21.89 | 24.14 | 30.79 | 53.07 | 43.85 | 45.97 |
| bpol | 9.00 | 4.00 | 1.00 | 2.00 | 1.00 | 2.00 | 2.00 | 3.00 | 1.00 | 2.00 | 2.00 | 4.00 | 10.00 | 6.00 | 7.00 |
| CASA- | 32.22 | 20.97 | 14.32 | 15.02 | 14.32 | 15.02 | 14.15 | 19.55 | 13.45 | 14.15 | 15.15 | 20.54 | 35.37 | 26.41 | 27.40 |
| CASA+ | 3135.47 | 1803.15 | 873.08 | 845.65 | 977.33 | 990.83 | 899.47 | 1777.21 | 911.73 | 916.25 | 568.62 | 1232.73 | 3314.88 | 2280.38 | 1705.84 |
| chi0 | 24.99 | 15.20 | 10.41 | 10.56 | 9.79 | 9.94 | 9.76 | 13.97 | 9.49 | 9.64 | 10.80 | 15.01 | 27.50 | 19.58 | 20.62 |
| chi0_C | 23.04 | 13.85 | 8.82 | 9.36 | 8.82 | 9.36 | 8.95 | 12.92 | 8.41 | 8.95 | 10.04 | 14.01 | 24.86 | 17.06 | 18.15 |
| chi0v | 20.64 | 10.98 | 6.96 | 6.67 | 5.96 | 5.67 | 5.75 | 9.64 | 6.04 | 5.75 | 6.25 | 10.14 | 22.30 | 14.14 | 14.64 |
| chi0v_C | 24.40 | 12.56 | 7.33 | 7.03 | 6.33 | 6.03 | 6.16 | 10.85 | 6.46 | 6.16 | 6.74 | 11.43 | 25.85 | 15.59 | 16.16 |
| chi1 | 15.02 | 8.51 | 5.80 | 5.95 | 5.49 | 5.64 | 5.57 | 8.14 | 5.39 | 5.53 | 6.29 | 8.87 | 16.42 | 10.79 | 11.52 |
| chi1_C | 2.00 | 2.00 | 5.00 | 5.00 | 5.00 | 5.00 | 5.00 | 5.00 | 5.00 | 5.00 | 5.00 | 5.00 | 5.00 | 5.00 | 5.00 |
| chi1v | 10.71 | 4.85 | 2.95 | 2.78 | 2.45 | 2.28 | 2.36 | 4.52 | 2.53 | 2.36 | 2.24 | 4.40 | 11.38 | 6.02 | 5.90 |
| chi1v_C | 15.02 | 6.53 | 3.23 | 3.06 | 2.65 | 2.48 | 2.65 | 5.80 | 2.82 | 2.65 | 2.41 | 5.56 | 15.45 | 7.53 | 7.29 |
| chiral | 2.00 | 2.00 | 5.00 | 5.00 | 5.00 | 5.00 | 5.00 | 5.00 | 5.00 | 5.00 | 5.00 | 5.00 | 5.00 | 5.00 | 5.00 |
| chiral_u | 148.87 | 33.35 | 27.19 | 34.14 | 35.52 | 34.41 | 30.89 | 79.26 | 15.93 | 11.80 | 141.66 | 124.72 | 140.36 | 12.46 | 98.28 |
| DASA | 1730.00 | 208.60 | 198.09 | 229.59 | 246.32 | 179.98 | 175.08 | 678.51 | 146.67 | 61.57 | 981.98 | 1089.58 | 1760.54 | 208.44 | 1035.22 |
| DCASA | 1.06 | 1.16 | 1.19 | 1.23 | 1.30 | 1.32 | 1.23 | 1.17 | 1.22 | 1.27 | 1.24 | 1.18 | 1.08 | 1.14 | 1.15 |
| dens | 0.75 | 0.84 | 0.87 | 0.89 | 0.94 | 0.97 | 0.90 | 0.84 | 0.89 | 0.92 | 0.89 | 0.84 | 0.76 | 0.83 | 0.83 |
| density | 19.00 | 16.00 | 9.00 | 9.00 | 9.00 | 9.00 | 9.00 | 14.00 | 9.00 | 9.00 | 9.00 | 14.00 | 19.00 | 16.00 | 16.00 |
| diameter | 3.31 | 0.52 | 1.73 | 1.78 | 1.74 | 1.09 | 1.63 | 2.06 | 1.71 | 1.14 | 2.56 | 2.63 | 3.18 | 2.87 | 3.22 |
| dipole | 1.52 | 0.18 | -0.43 | -0.55 | -0.47 | 0.23 | -1.25 | -1.01 | -0.40 | -0.14 | 0.68 | 2.38 | 1.94 | 2.75 | 2.56 |
| dipoleX | -2.76 | 0.19 | -0.78 | -0.84 | -0.79 | 0.78 | 0.66 | -1.32 | -0.74 | 0.77 | -1.90 | 0.85 | -2.44 | 0.55 | -1.83 |
| dipoleY | 1.02 | 0.44 | -1.48 | -1.47 | -1.48 | -0.73 | -0.81 | 1.22 | -1.48 | -0.83 | -1.57 | -0.72 | 0.63 | 0.64 | 0.69 |
| dipoleZ | 159.42 | 38.11 | 33.70 | 38.62 | 31.56 | 26.26 | 24.40 | 101.45 | 31.19 | 14.70 | 44.55 | 71.93 | 198.87 | 82.15 | 76.64 |
| E | 16.58 | 17.95 | 5.95 | 6.15 | 6.66 | 6.84 | 11.51 | 23.19 | 5.90 | 5.98 | 10.94 | 8.99 | 45.55 | 27.44 | 12.94 |
| E_ang | 20.65 | -38.77 | -8.98 | -8.63 | -8.87 | -20.37 | -28.02 | -36.38 | -8.65 | -31.37 | -20.01 | -13.18 | -18.53 | -55.35 | -3.46 |
| E_ele | 120.47 | 9.81 | 18.44 | 20.61 | 14.67 | 6.67 | 0.36 | 13.72 | 15.08 | -3.98 | 21.01 | 44.40 | 102.49 | -6.07 | 49.48 |
| E_nb | 0.25 | 0.22 | 0.01 | 0.01 | 0.01 | 0.01 | 0.01 | 21.22 | 0.01 | 0.01 | 0.07 | 0.09 | 0.20 | 21.22 | 0.04 |
| E_oop | 0.00 | 0.00 | 0.00 | 0.00 | 0.00 | 0.00 | 0.00 | 0.00 | 0.00 | 0.00 | 0.00 | 0.00 | 0.00 | 0.00 | 0.00 |
| E_rele | 0.00 | 0.00 | 0.00 | 0.00 | 0.00 | 0.00 | 0.00 | 0.00 | 0.00 | 0.00 | 0.00 | 0.00 | 0.00 | 0.00 | 0.00 |
| E_rnb | 0.00 | 0.00 | 0.00 | 0.00 | 0.00 | 0.00 | 0.00 | 0.00 | 0.00 | 0.00 | 0.00 | 0.00 | 0.00 | 0.00 | 0.00 |
| E_rsol | 0.00 | 0.00 | 0.00 | 0.00 | 0.00 | 0.00 | 0.00 | 0.00 | 0.00 | 0.00 | 0.00 | 0.00 | 0.00 | 0.00 | 0.00 |
| E_rvdw | -80.09 | -14.82 | -27.10 | -27.79 | -26.61 | -10.03 | -23.22 | -44.42 | -27.35 | -9.94 | -83.20 | -73.49 | -53.51 | -35.33 | -92.16 |
| E_sol | 1.66 | 0.14 | 0.31 | 0.32 | 0.32 | 0.34 | 0.31 | -10.62 | 0.30 | 0.41 | 0.23 | 1.33 | -0.02 | -10.73 | 1.20 |
| E_stb | 13.82 | 4.14 | 2.35 | 2.44 | 2.34 | 2.51 | 2.81 | 33.14 | 2.34 | 2.39 | 3.65 | 5.01 | 33.19 | 32.99 | 4.40 |
| E_str | 0.02 | 0.01 | 5.00 | 6.47 | 2.10 | 0.00 | 0.00 | 60.83 | 2.32 | 0.00 | 0.00 | 0.01 | 48.25 | 50.96 | 0.03 |
| E_strain | 6.64 | 5.85 | 6.64 | 9.09 | 7.55 | 9.90 | 9.40 | 20.81 | 7.55 | 9.90 | 8.66 | 12.11 | 17.45 | 17.30 | 8.58 |
| E_tor | 99.83 | 48.58 | 27.42 | 29.24 | 23.55 | 27.04 | 28.38 | 50.10 | 23.73 | 27.40 | 41.02 | 57.59 | 121.02 | 49.28 | 52.94 |
| E_vdw | 0.57 | 0.39 | 0.30 | 0.27 | 0.35 | 0.35 | 0.42 | 0.32 | 0.37 | 0.37 | 0.53 | 0.52 | 0.50 | 0.34 | 0.42 |
| FASA- | 0.81 | 0.68 | 0.43 | 0.48 | 0.39 | 0.38 | 0.36 | 0.63 | 0.38 | 0.37 | 0.38 | 0.58 | 0.83 | 0.69 | 0.68 |
| FASA_H | 0.19 | 0.32 | 0.57 | 0.52 | 0.61 | 0.62 | 0.64 | 0.37 | 0.62 | 0.63 | 0.62 | 0.42 | 0.17 | 0.31 | 0.32 |
| FASA_P | 5.09 | 2.43 | 1.47 | 1.30 | 1.67 | 1.83 | 2.40 | 1.82 | 1.77 | 1.95 | 3.29 | 3.67 | 4.98 | 2.66 | 3.40 |
| FASA+ | 0.41 | 0.44 | 0.36 | 0.34 | 0.43 | 0.43 | 0.35 | 0.46 | 0.40 | 0.40 | 0.23 | 0.32 | 0.37 | 0.36 | 0.30 |
| FCASA- | 1.00 | 0.00 | 0.00 | 0.00 | 0.00 | 0.00 | 0.00 | 0.00 | 0.00 | 0.00 | 1.00 | 1.00 | 1.00 | 0.00 | 1.00 |
| FCASA+ | 3.28 | 2.75 | 1.90 | 1.79 | 2.23 | 2.23 | 2.01 | 2.94 | 2.11 | 2.09 | 1.21 | 1.95 | 3.25 | 2.93 | 2.11 |
| FCharge | -0.78 | -0.79 | -0.79 | -0.79 | -0.79 | -0.79 | -0.84 | -0.79 | -0.79 | -0.79 | -0.78 | -0.79 | -0.78 | -0.79 | -0.78 |
| GCUT_PEOE_0 | -0.39 | -0.45 | -0.42 | -0.44 | -0.47 | -0.46 | -0.43 | -0.43 | -0.45 | -0.45 | -0.40 | -0.40 | -0.39 | -0.44 | -0.41 |
| GCUT_PEOE_1 | 0.08 | 0.08 | 0.10 | 0.10 | 0.10 | 0.11 | 0.14 | 0.11 | 0.10 | 0.11 | 0.15 | 0.13 | 0.08 | 0.08 | 0.09 |
| GCUT_PEOE_2 | 2.93 | 2.54 | 2.65 | 2.66 | 2.61 | 2.63 | 2.62 | 2.67 | 2.60 | 2.62 | 2.71 | 2.76 | 3.08 | 2.83 | 2.90 |
| GCUT_PEOE_3 | -1.42 | -1.17 | -1.17 | -1.17 | -1.17 | -1.17 | -1.37 | -1.17 | -1.17 | -1.17 | -1.42 | -1.42 | -1.42 | -1.17 | -1.42 |
| GCUT_SLOGP_0 | -0.26 | -0.32 | -0.43 | -0.44 | -0.43 | -0.45 | -0.49 | -0.44 | -0.43 | -0.45 | -0.47 | -0.42 | -0.27 | -0.33 | -0.31 |
| GCUT_SLOGP_1 | 0.16 | 0.16 | 0.07 | 0.05 | 0.08 | 0.08 | 0.10 | 0.15 | 0.07 | 0.05 | 0.08 | 0.15 | 0.16 | 0.14 | 0.13 |
| GCUT_SLOGP_2 | 3.00 | 2.48 | 2.55 | 2.55 | 2.50 | 2.50 | 2.50 | 2.58 | 2.50 | 2.49 | 2.62 | 2.70 | 3.11 | 2.74 | 2.84 |
| GCUT_SLOGP_3 | -0.55 | -0.51 | -0.52 | -0.52 | -0.52 | -0.52 | -0.52 | -0.52 | -0.52 | -0.52 | -0.55 | -0.55 | -0.55 | -0.52 | -0.55 |
| GCUT_SMR_0 | -0.22 | -0.22 | -0.22 | -0.23 | -0.22 | -0.23 | -0.24 | -0.23 | -0.22 | -0.23 | -0.24 | -0.23 | -0.22 | -0.22 | -0.22 |
| GCUT_SMR_1 | 0.19 | 0.18 | 0.14 | 0.15 | 0.15 | 0.18 | 0.18 | 0.21 | 0.15 | 0.18 | 0.19 | 0.21 | 0.19 | 0.18 | 0.19 |
| GCUT_SMR_2 | 3.19 | 2.79 | 2.85 | 2.87 | 2.81 | 2.82 | 2.82 | 2.89 | 2.81 | 2.82 | 2.89 | 2.96 | 3.32 | 3.04 | 3.10 |
| GCUT_SMR_3 | 0.13 | 0.04 | 0.17 | 0.17 | 0.15 | 0.14 | 0.10 | 0.05 | 0.15 | 0.14 | 0.03 | 0.02 | 0.13 | 0.05 | 0.06 |
| glob | 35.40 | 23.66 | 15.39 | 16.37 | 15.39 | 16.37 | 15.39 | 21.70 | 14.41 | 15.39 | 15.88 | 22.20 | 39.24 | 30.54 | 30.95 |
| Kier1 | 16.56 | 11.04 | 5.78 | 6.41 | 5.78 | 6.41 | 6.19 | 9.67 | 5.55 | 6.19 | 6.25 | 9.65 | 18.30 | 14.40 | 14.25 |
| Kier2 | 7.78 | 6.49 | 2.88 | 3.12 | 2.88 | 3.12 | 3.03 | 4.95 | 2.80 | 3.03 | 2.70 | 4.48 | 8.71 | 8.76 | 7.85 |
| Kier3 | 24.77 | 17.19 | 12.81 | 13.76 | 12.68 | 13.62 | 13.19 | 17.12 | 11.86 | 12.81 | 13.19 | 17.22 | 28.79 | 23.62 | 23.65 |
| KierA1 | 11.49 | 7.92 | 4.76 | 5.33 | 4.71 | 5.28 | 5.25 | 7.55 | 4.51 | 5.09 | 5.14 | 7.41 | 13.33 | 11.05 | 10.81 |
| KierA2 | 5.37 | 4.62 | 2.35 | 2.58 | 2.32 | 2.55 | 2.55 | 3.84 | 2.26 | 2.47 | 2.20 | 3.42 | 6.32 | 6.68 | 5.92 |
| KierA3 | 6.06 | 4.70 | 3.21 | 3.67 | 3.14 | 3.59 | 3.65 | 4.79 | 2.97 | 3.43 | 3.23 | 4.40 | 7.53 | 7.25 | 6.73 |
| KierFlex | 9.00 | 9.00 | 7.00 | 8.00 | 7.00 | 8.00 | 8.00 | 9.00 | 7.00 | 8.00 | 9.00 | 10.00 | 11.00 | 12.00 | 13.00 |
| lip_acc | 2.00 | 1.00 | 3.00 | 4.00 | 3.00 | 4.00 | 5.00 | 4.00 | 3.00 | 4.00 | 6.00 | 5.00 | 3.00 | 1.00 | 2.00 |
| lip_don | 0.00 | 1.00 | 1.00 | 1.00 | 1.00 | 1.00 | 1.00 | 1.00 | 1.00 | 1.00 | 1.00 | 1.00 | 0.00 | 0.00 | 0.00 |
| lip_druglike | 2.00 | 0.00 | 0.00 | 0.00 | 0.00 | 0.00 | 0.00 | 0.00 | 0.00 | 0.00 | 1.00 | 0.00 | 3.00 | 2.00 | 2.00 |
| lip_violation | 7.54 | 1.26 | -1.53 | -2.57 | -1.35 | -2.38 | -2.29 | -0.67 | -1.58 | -2.61 | -1.59 | 0.04 | 6.79 | 1.10 | 1.80 |
| logP(o/w) | -10.47 | -4.37 | -0.74 | -0.21 | -1.11 | -0.58 | -0.40 | -2.45 | -0.73 | -0.20 | -1.26 | -3.51 | -9.78 | -4.30 | -5.37 |
| logS | 13.92 | 4.00 | 5.18 | 5.04 | 4.79 | 5.29 | 7.25 | 2.59 | 5.20 | 5.86 | 10.04 | 1.28 | 11.37 | 6.96 | 9.02 |
| MNDO_dipole | -182916.97 | -131158.66 | -95114.16 | -102544.65 | -102231.31 | -109662.66 | -96605.08 | -125953.20 | -91504.14 | -98935.52 | -101834.23 | -131196.20 | -205667.41 | -167883.63 | -173124.86 |
| MNDO_E | -1766599.90 | -896111.94 | -575263.88 | -626590.00 | -583612.31 | -634875.81 | -575102.06 | -861813.31 | -529079.63 | -578877.31 | -648068.44 | -946647.19 | -2128350.30 | -1345511.10 | -1438441.30 |
| MNDO_Eele | 187.60 | -161.32 | -235.31 | -279.03 | -273.68 | -315.23 | -221.33 | -225.07 | -231.35 | -272.93 | 4.80 | -12.83 | 86.77 | -327.82 | -113.90 |
| MNDO_HF | -10.80 | -9.59 | -9.72 | -9.74 | -9.86 | -9.91 | -9.52 | -9.50 | -9.88 | -9.92 | -13.26 | -12.04 | -10.76 | -9.55 | -12.02 |
| MNDO_HOMO | 10.80 | 9.59 | 9.72 | 9.74 | 9.86 | 9.91 | 9.52 | 9.50 | 9.88 | 9.92 | 13.26 | 12.04 | 10.76 | 9.55 | 12.02 |
| MNDO_IP | -4.97 | -0.82 | -0.32 | -0.34 | -0.67 | -0.71 | -0.42 | -0.56 | -0.25 | -0.30 | -5.02 | -4.85 | -4.73 | -0.58 | -4.92 |
| MNDO_LUMO | 17.76 | 9.95 | 6.19 | 6.35 | 5.77 | 5.92 | 6.03 | 9.15 | 5.73 | 5.89 | 6.87 | 10.00 | 19.07 | 12.28 | 13.14 |
| mr | 1.00 | 1.00 | 1.00 | 1.00 | 1.00 | 1.00 | 1.00 | 1.00 | 1.00 | 1.00 | 1.00 | 1.00 | 1.00 | 1.00 | 1.00 |
|  |  |  |  |  |  |  |  |  |  |  |  |  |  |  |  |
| nmol | 0.26 | 0.12 | 0.28 | 0.28 | 0.28 | 0.28 | 0.25 | 0.11 | 0.25 | 0.26 | 0.20 | 0.13 | 0.29 | 0.17 | 0.19 |
| npr1 | 0.93 | 0.94 | 0.93 | 0.91 | 0.93 | 0.92 | 0.88 | 0.96 | 0.91 | 0.88 | 0.82 | 0.89 | 0.89 | 0.89 | 0.90 |
| npr2 | 40.00 | 18.00 | 12.00 | 12.00 | 12.00 | 12.00 | 12.00 | 18.00 | 12.00 | 12.00 | 16.00 | 22.00 | 40.00 | 18.00 | 22.00 |
| opr_brigid | 0.00 | 1.00 | 1.00 | 1.00 | 1.00 | 1.00 | 1.00 | 1.00 | 1.00 | 1.00 | 0.00 | 1.00 | 0.00 | 0.00 | 0.00 |
| opr_leadlike | 7.00 | 3.00 | 2.00 | 2.00 | 2.00 | 2.00 | 2.00 | 3.00 | 2.00 | 2.00 | 3.00 | 4.00 | 7.00 | 3.00 | 4.00 |
| opr_nring | 10.00 | 6.00 | 3.00 | 4.00 | 3.00 | 4.00 | 4.00 | 6.00 | 3.00 | 4.00 | 4.00 | 6.00 | 12.00 | 9.00 | 9.00 |
| opr_nrot | 4.00 | 1.00 | 0.00 | 0.00 | 0.00 | 0.00 | 0.00 | 1.00 | 0.00 | 0.00 | 2.00 | 1.00 | 5.00 | 2.00 | 2.00 |
| opr_violation | 8.99 | 6.26 | 4.83 | 4.83 | 4.83 | 5.23 | 5.67 | 5.61 | 4.83 | 5.22 | 6.15 | 7.12 | 9.89 | 7.77 | 8.08 |
| PC- | 4.64 | 2.96 | 2.49 | 2.84 | 2.65 | 3.00 | 2.83 | 3.30 | 2.46 | 2.81 | 3.78 | 4.30 | 5.35 | 3.85 | 4.85 |
| PC+ | -7.99 | -6.26 | -5.23 | -5.23 | -5.23 | -5.23 | -5.67 | -6.46 | -5.23 | -5.22 | -5.15 | -6.12 | -8.89 | -8.17 | -7.08 |
| PEOE_PC- | 0.07 | 0.12 | 0.13 | 0.12 | 0.13 | 0.11 | 0.12 | 0.11 | 0.13 | 0.12 | 0.06 | 0.08 | 0.06 | 0.09 | 0.07 |
| PEOE_PC+ | -3.64 | -2.96 | -2.49 | -2.84 | -2.65 | -3.00 | -2.83 | -3.30 | -2.46 | -2.81 | -2.78 | -3.30 | -4.35 | -3.85 | -3.85 |
| PEOE_RPC- | 12.25 | 50.50 | 44.55 | 31.75 | 12.80 | 0.00 | 16.75 | 18.95 | 41.80 | 29.00 | 0.00 | 0.00 | 31.54 | 113.59 | 94.63 |
| PEOE_RPC+ | 0.10 | 0.11 | 0.16 | 0.14 | 0.15 | 0.13 | 0.14 | 0.12 | 0.16 | 0.14 | 0.14 | 0.12 | 0.09 | 0.09 | 0.08 |
| PEOE_VSA_FHYD | 0.53 | 0.43 | 0.37 | 0.32 | 0.36 | 0.31 | 0.30 | 0.41 | 0.32 | 0.26 | 0.21 | 0.34 | 0.49 | 0.35 | 0.30 |
| PEOE_VSA_FNEG | 0.05 | 0.19 | 0.23 | 0.25 | 0.24 | 0.26 | 0.23 | 0.19 | 0.24 | 0.26 | 0.17 | 0.10 | 0.06 | 0.19 | 0.13 |
| PEOE_VSA_FPNEG | 0.18 | 0.36 | 0.43 | 0.48 | 0.49 | 0.54 | 0.42 | 0.36 | 0.46 | 0.51 | 0.46 | 0.38 | 0.21 | 0.34 | 0.35 |
| PEOE_VSA_FPOL | 0.47 | 0.57 | 0.63 | 0.68 | 0.64 | 0.69 | 0.70 | 0.59 | 0.68 | 0.74 | 0.79 | 0.66 | 0.51 | 0.65 | 0.70 |
| PEOE_VSA_FPOS | 0.13 | 0.17 | 0.20 | 0.23 | 0.25 | 0.28 | 0.19 | 0.18 | 0.22 | 0.25 | 0.29 | 0.27 | 0.14 | 0.15 | 0.23 |
| PEOE_VSA_FPPOS | 477.64 | 241.84 | 145.83 | 137.77 | 123.99 | 115.93 | 146.03 | 222.22 | 128.60 | 120.54 | 144.60 | 225.74 | 511.71 | 316.85 | 320.37 |
| PEOE_VSA_HYD | 307.41 | 162.79 | 94.71 | 83.73 | 87.87 | 76.89 | 75.87 | 144.20 | 75.97 | 64.99 | 55.87 | 124.20 | 317.68 | 169.11 | 149.10 |
| PEOE_VSA_NEG | 30.76 | 71.32 | 57.22 | 64.99 | 57.22 | 64.99 | 57.93 | 64.99 | 57.22 | 64.99 | 44.50 | 37.99 | 41.03 | 89.90 | 62.90 |
| PEOE_VSA_PNEG | 107.88 | 134.27 | 108.26 | 126.35 | 117.28 | 135.37 | 106.35 | 126.35 | 108.26 | 126.35 | 122.57 | 137.63 | 134.72 | 164.41 | 175.69 |
| PEOE_VSA_POL | 278.10 | 213.32 | 159.37 | 180.38 | 153.39 | 174.40 | 176.51 | 204.37 | 160.89 | 181.90 | 211.30 | 239.17 | 328.75 | 312.16 | 346.95 |
| PEOE_VSA_POS | 77.12 | 62.95 | 51.04 | 61.36 | 60.06 | 70.38 | 48.41 | 61.36 | 51.04 | 61.36 | 78.07 | 99.64 | 93.70 | 74.52 | 112.79 |
| PEOE_VSA_PPOS | 0.47 | 0.50 | 0.44 | 0.44 | 0.44 | 0.44 | 0.44 | 0.50 | 0.44 | 0.44 | 0.44 | 0.50 | 0.47 | 0.50 | 0.50 |
| PEOE_VSA+0 | 119.53 | 66.14 | 30.50 | 49.07 | 47.24 | 65.82 | 53.34 | 74.67 | 34.76 | 53.34 | 40.54 | 64.08 | 128.06 | 74.67 | 64.08 |
| PEOE_VSA+1 | 12.80 | 16.10 | 9.81 | 14.71 | 9.81 | 14.71 | 25.91 | 25.91 | 9.81 | 14.71 | 27.51 | 27.51 | 22.61 | 25.91 | 22.61 |
| PEOE_VSA+2 | 56.40 | 17.64 | 23.48 | 23.48 | 23.48 | 23.48 | 32.10 | 23.48 | 23.48 | 23.48 | 65.18 | 47.94 | 52.85 | 23.48 | 52.85 |
| PEOE_VSA+3 | 64.17 | 17.85 | 20.65 | 30.97 | 29.67 | 39.99 | 30.97 | 30.97 | 20.65 | 30.97 | 78.07 | 86.69 | 66.04 | 0.00 | 55.71 |
| PEOE_VSA+4 | 0.00 | 27.66 | 12.95 | 12.95 | 12.95 | 12.95 | 0.00 | 12.95 | 12.95 | 12.95 | 0.00 | 0.00 | 14.71 | 57.08 | 44.13 |
| PEOE_VSA+5 | 12.95 | 17.44 | 17.44 | 17.44 | 17.44 | 17.44 | 17.44 | 17.44 | 17.44 | 17.44 | 0.00 | 12.95 | 12.95 | 17.44 | 12.95 |
| PEOE_VSA+6 | 31.14 | 49.02 | 37.50 | 18.75 | 18.75 | 0.00 | 12.25 | 36.76 | 18.75 | 0.00 | 0.00 | 24.51 | 31.14 | 36.76 | 24.51 |
| PEOE_VSA-0 | 220.59 | 36.76 | 0.00 | 0.00 | 0.00 | 0.00 | 0.00 | 36.76 | 0.00 | 0.00 | 0.00 | 36.76 | 220.59 | 36.76 | 36.76 |
| PEOE_VSA-1 | 0.00 | 0.00 | 0.00 | 0.00 | 0.00 | 0.00 | 0.00 | 0.00 | 0.00 | 0.00 | 0.00 | 0.00 | 0.00 | 0.00 | 0.00 |
| PEOE_VSA-2 | 24.93 | 5.68 | 0.00 | 0.00 | 11.91 | 11.91 | 5.68 | 5.68 | 0.00 | 0.00 | 11.37 | 24.93 | 24.93 | 5.68 | 24.93 |
| PEOE_VSA-3 | 11.91 | 25.48 | 25.48 | 25.48 | 25.48 | 25.48 | 25.48 | 25.48 | 25.48 | 25.48 | 18.56 | 11.91 | 11.91 | 25.48 | 11.91 |
| PEOE_VSA-4 | 16.34 | 40.70 | 13.70 | 13.70 | 13.70 | 13.70 | 0.00 | 13.57 | 13.70 | 13.70 | 2.64 | 0.27 | 13.84 | 54.27 | 40.97 |
| PEOE_VSA-5 | 2.50 | 5.14 | 18.04 | 25.81 | 18.04 | 25.81 | 32.46 | 25.94 | 18.04 | 25.81 | 23.30 | 25.81 | 15.28 | 10.15 | 10.02 |
| PEOE_VSA-6 | 0.82 | 0.64 | 0.57 | 0.52 | 0.51 | 0.46 | 0.58 | 0.64 | 0.54 | 0.49 | 0.54 | 0.62 | 0.79 | 0.66 | 0.65 |
| petitjean | 0.90 | 1.00 | 0.80 | 0.80 | 0.80 | 0.80 | 0.80 | 1.00 | 0.80 | 0.80 | 0.80 | 1.00 | 0.90 | 1.00 | 1.00 |
| petitjeanSC | 14.96 | 3.21 | 5.23 | 5.82 | 4.99 | 5.26 | 6.57 | 3.18 | 5.31 | 5.80 | 10.56 | 3.60 | 12.20 | 7.02 | 9.14 |
| PM3_dipole | -167082.05 | -119571.80 | -86910.66 | -93680.02 | -93253.38 | -100024.21 | -87544.82 | -114846.35 | -83458.95 | -90229.85 | -91181.44 | -118483.63 | -188264.42 | -153694.63 | -157329.17 |
| PM3_E | -1746820.50 | -881546.13 | -566095.63 | -616373.38 | -572034.69 | -624520.00 | -565556.06 | -848255.06 | -519778.47 | -570822.00 | -636095.69 | -929103.25 | -2117933.50 | -1338256.10 | -1424898.60 |
| PM3_Eele | 175.14 | -189.02 | -248.45 | -285.61 | -279.25 | -318.39 | -224.44 | -228.13 | -238.48 | -278.75 | -8.19 | -8.32 | 49.99 | -343.49 | -96.45 |
| PM3_HF | -11.07 | -9.35 | -9.40 | -9.40 | -9.69 | -9.79 | -9.29 | -9.37 | -9.66 | -9.76 | -12.90 | -12.36 | -11.04 | -9.30 | -12.40 |
| PM3_HOMO | 11.07 | 9.35 | 9.40 | 9.40 | 9.69 | 9.79 | 9.29 | 9.37 | 9.66 | 9.76 | 12.90 | 12.36 | 11.04 | 9.30 | 12.40 |
| PM3_IP | -5.15 | -0.83 | -0.38 | -0.42 | -0.72 | -0.81 | -0.54 | -0.80 | -0.41 | -0.50 | -4.96 | -4.92 | -4.76 | -0.72 | -5.08 |
| PM3_LUMO | 21184.97 | 10315.67 | 2864.42 | 3282.16 | 2906.57 | 3321.79 | 2962.90 | 8757.14 | 2577.71 | 2965.41 | 3674.33 | 9558.40 | 22884.60 | 14040.52 | 15724.53 |
| pmi | 4993.68 | 1229.81 | 722.58 | 850.94 | 729.90 | 849.99 | 702.88 | 909.55 | 601.29 | 714.91 | 719.87 | 1205.10 | 6185.61 | 2337.80 | 2811.55 |
| pmi1 | 17976.44 | 9398.75 | 2415.17 | 2723.03 | 2453.53 | 2768.34 | 2448.48 | 8152.71 | 2168.65 | 2447.37 | 2991.02 | 8435.93 | 18598.48 | 12143.72 | 13581.22 |
| pmi2 | 19399.82 | 10002.78 | 2591.08 | 2990.35 | 2629.71 | 3025.26 | 2774.44 | 8452.01 | 2385.48 | 2768.54 | 3637.77 | 9475.75 | 20985.11 | 13599.52 | 15056.28 |
| pmi3 | 5511.15 | 3889.53 | 955.88 | 997.81 | 992.82 | 1039.19 | 734.52 | 3598.12 | 695.64 | 747.92 | 1057.22 | 4561.93 | 7034.18 | 4632.57 | 6151.80 |
| pmiX | 13606.09 | 5435.97 | 1552.49 | 1800.51 | 1559.32 | 1808.39 | 1747.08 | 4493.88 | 1527.77 | 1761.94 | 2165.81 | 4569.16 | 13877.95 | 7826.16 | 7812.02 |
| pmiY | 2067.72 | 990.17 | 356.04 | 483.84 | 354.43 | 474.21 | 481.31 | 665.14 | 354.30 | 455.54 | 451.30 | 427.30 | 1972.47 | 1581.80 | 1760.70 |
| pmiZ | 8.99 | 6.26 | 4.83 | 4.83 | 4.83 | 5.23 | 5.67 | 5.61 | 4.83 | 5.22 | 6.15 | 7.12 | 9.89 | 7.77 | 8.08 |
| Q_PC- | 0.08 | 0.13 | 0.14 | 0.14 | 0.14 | 0.13 | 0.15 | 0.15 | 0.14 | 0.13 | 0.09 | 0.08 | 0.07 | 0.11 | 0.08 |
| Q_PC+ | -7.99 | -6.26 | -5.23 | -5.23 | -5.23 | -5.23 | -5.67 | -6.46 | -5.23 | -5.22 | -5.15 | -6.12 | -8.89 | -8.17 | -7.08 |
| Q_RPC- | 0.72 | 0.52 | 0.33 | 0.36 | 0.30 | 0.29 | 0.27 | 0.53 | 0.28 | 0.27 | 0.27 | 0.43 | 0.70 | 0.51 | 0.50 |
| Q_RPC+ | 0.08 | 0.11 | 0.13 | 0.13 | 0.13 | 0.13 | 0.15 | 0.13 | 0.13 | 0.13 | 0.17 | 0.11 | 0.07 | 0.10 | 0.09 |
| Q_VSA_FHYD | 0.55 | 0.48 | 0.38 | 0.38 | 0.37 | 0.37 | 0.37 | 0.47 | 0.35 | 0.38 | 0.21 | 0.34 | 0.49 | 0.39 | 0.30 |
| Q_VSA_FNEG | 0.10 | 0.21 | 0.24 | 0.25 | 0.25 | 0.26 | 0.25 | 0.20 | 0.26 | 0.26 | 0.21 | 0.17 | 0.10 | 0.20 | 0.18 |
| Q_VSA_FPNEG | 0.28 | 0.48 | 0.67 | 0.64 | 0.70 | 0.71 | 0.73 | 0.47 | 0.72 | 0.73 | 0.73 | 0.57 | 0.30 | 0.49 | 0.50 |
| Q_VSA_FPOL | 0.45 | 0.52 | 0.62 | 0.62 | 0.63 | 0.63 | 0.63 | 0.53 | 0.65 | 0.62 | 0.79 | 0.66 | 0.51 | 0.61 | 0.70 |
| Q_VSA_FPOS | 0.18 | 0.27 | 0.42 | 0.39 | 0.45 | 0.46 | 0.48 | 0.27 | 0.46 | 0.46 | 0.53 | 0.40 | 0.20 | 0.29 | 0.32 |
| Q_VSA_FPPOS | 422.59 | 196.14 | 84.62 | 94.95 | 71.80 | 71.80 | 67.39 | 183.17 | 67.39 | 67.39 | 70.95 | 155.76 | 452.06 | 246.83 | 250.39 |
| Q_VSA_HYD | 319.67 | 179.53 | 96.98 | 100.48 | 90.14 | 93.64 | 92.62 | 163.15 | 82.49 | 93.99 | 55.87 | 124.20 | 317.68 | 188.06 | 149.10 |
| Q_VSA_NEG | 55.90 | 77.35 | 61.48 | 64.99 | 61.48 | 64.99 | 63.62 | 70.67 | 61.48 | 64.99 | 55.73 | 62.78 | 65.82 | 95.58 | 87.69 |
| Q_VSA_PNEG | 162.92 | 179.97 | 169.47 | 169.17 | 169.47 | 179.50 | 184.99 | 165.40 | 169.47 | 179.50 | 196.22 | 207.60 | 194.38 | 234.43 | 245.66 |
| Q_VSA_POL | 265.85 | 196.58 | 157.11 | 163.64 | 151.13 | 157.65 | 159.76 | 185.42 | 154.36 | 152.90 | 211.30 | 239.17 | 328.75 | 293.20 | 346.95 |
| Q_VSA_POS | 107.03 | 102.62 | 107.98 | 104.19 | 107.98 | 114.51 | 121.37 | 94.73 | 107.98 | 114.51 | 140.49 | 144.82 | 128.55 | 138.85 | 157.97 |
| Q_VSA_PPOS | 10.00 | 8.00 | 5.00 | 5.00 | 5.00 | 5.00 | 5.00 | 7.00 | 5.00 | 5.00 | 5.00 | 7.00 | 10.00 | 8.00 | 8.00 |
| radius | 1.00 | 1.00 | 0.00 | 0.00 | 0.00 | 0.00 | 0.00 | 0.00 | 0.00 | 0.00 | 0.00 | 0.00 | 0.00 | 0.00 | 0.00 |
| reactive | 5.81 | 5.07 | 3.23 | 3.36 | 3.23 | 3.36 | 3.28 | 4.80 | 3.15 | 3.28 | 3.50 | 4.86 | 5.76 | 5.27 | 5.44 |
| rgyr | 7.00 | 3.00 | 2.00 | 2.00 | 2.00 | 2.00 | 2.00 | 3.00 | 2.00 | 2.00 | 3.00 | 4.00 | 7.00 | 3.00 | 4.00 |
| rings | 0.08 | 0.13 | 0.14 | 0.14 | 0.14 | 0.13 | 0.15 | 0.15 | 0.14 | 0.13 | 0.09 | 0.08 | 0.07 | 0.11 | 0.08 |
| RPC- | 1.00 | 0.76 | 1.00 | 1.00 | 1.00 | 1.00 | 1.00 | 1.00 | 1.00 | 1.00 | 1.00 | 1.00 | 1.00 | 1.00 | 1.00 |
| RPC+ | 0.08 | 0.11 | 0.13 | 0.13 | 0.13 | 0.13 | 0.15 | 0.13 | 0.13 | 0.13 | 0.17 | 0.11 | 0.07 | 0.10 | 0.09 |
| rsynth | 6.23 | 1.47 | -0.75 | -1.78 | -0.85 | -1.87 | -1.93 | -0.46 | -1.14 | -2.17 | -2.13 | -0.46 | 5.44 | 1.25 | 1.26 |
| SlogP | 16.79 | 18.01 | 68.78 | 94.17 | 68.78 | 94.17 | 109.05 | 94.17 | 68.78 | 94.17 | 125.84 | 92.94 | 42.17 | 18.01 | 16.79 |
| SlogP_VSA0 | 64.43 | 28.97 | 13.21 | 13.21 | 13.21 | 13.21 | 10.28 | 15.54 | 13.21 | 13.21 | 35.47 | 58.74 | 66.49 | 38.78 | 81.98 |
| SlogP_VSA1 | 52.33 | 82.23 | 70.65 | 70.65 | 70.65 | 70.65 | 46.79 | 46.79 | 70.65 | 70.65 | 40.75 | 40.75 | 86.61 | 151.38 | 145.34 |
| SlogP_VSA2 | 20.93 | 20.93 | 0.00 | 20.93 | 0.00 | 20.93 | 20.93 | 20.93 | 0.00 | 20.93 | 20.93 | 20.93 | 20.93 | 20.93 | 20.93 |
| SlogP_VSA3 | 36.60 | 27.05 | 0.00 | 0.00 | 0.00 | 0.00 | 0.00 | 27.05 | 0.00 | 0.00 | 0.00 | 27.05 | 36.60 | 27.05 | 27.05 |
| SlogP_VSA4 | 9.12 | 25.91 | 3.19 | 3.19 | 9.12 | 9.12 | 16.79 | 16.79 | 0.00 | 0.00 | 0.00 | 0.00 | 0.00 | 16.79 | 0.00 |
| SlogP_VSA5 | 0.00 | 0.00 | 0.00 | 0.00 | 0.00 | 0.00 | 0.00 | 0.00 | 0.00 | 0.00 | 0.00 | 0.00 | 0.00 | 0.00 | 0.00 |
| SlogP_VSA6 | 407.94 | 140.72 | 17.21 | 17.21 | 17.21 | 17.21 | 34.86 | 123.07 | 34.86 | 34.86 | 37.43 | 125.65 | 390.29 | 123.07 | 125.65 |
| SlogP_VSA7 | 6.04 | 0.00 | 0.00 | 0.00 | 0.00 | 0.00 | 0.00 | 0.00 | 0.00 | 0.00 | 6.04 | 6.04 | 6.04 | 0.00 | 6.04 |
| SlogP_VSA8 | 17.29 | 72.15 | 105.47 | 72.15 | 87.11 | 53.78 | 38.82 | 38.82 | 72.15 | 38.82 | 17.29 | 17.29 | 50.61 | 138.80 | 117.27 |
| SlogP_VSA9 | 17.20 | 9.72 | 6.04 | 6.18 | 5.58 | 5.72 | 6.07 | 9.04 | 5.58 | 5.72 | 6.65 | 9.65 | 18.42 | 11.90 | 12.51 |
| SMR | 86.51 | 117.45 | 58.72 | 58.72 | 58.72 | 58.72 | 34.86 | 58.72 | 58.72 | 58.72 | 27.79 | 51.65 | 97.51 | 163.31 | 156.23 |
| SMR_VSA0 | 50.43 | 34.87 | 68.85 | 94.24 | 83.81 | 109.20 | 111.03 | 111.03 | 68.85 | 94.24 | 126.59 | 126.59 | 75.82 | 34.87 | 50.43 |
| SMR_VSA1 | 0.00 | 18.01 | 18.01 | 18.01 | 18.01 | 18.01 | 0.00 | 18.01 | 18.01 | 18.01 | 0.00 | 0.00 | 0.00 | 18.01 | 0.00 |
| SMR_VSA2 | 34.37 | 24.82 | 38.98 | 38.98 | 44.91 | 44.91 | 35.79 | 38.98 | 35.79 | 35.79 | 35.79 | 38.98 | 48.53 | 38.98 | 38.98 |
| SMR_VSA3 | 24.20 | 0.00 | 0.00 | 0.00 | 0.00 | 0.00 | 32.90 | 0.00 | 0.00 | 0.00 | 6.19 | 24.20 | 24.20 | 0.00 | 24.20 |
| SMR_VSA4 | 404.08 | 140.72 | 17.21 | 17.21 | 17.21 | 17.21 | 34.86 | 123.07 | 34.86 | 34.86 | 66.47 | 121.79 | 386.43 | 123.07 | 121.79 |
| SMR_VSA5 | 31.87 | 46.78 | 10.09 | 31.01 | 10.09 | 31.01 | 28.08 | 33.34 | 10.09 | 31.01 | 20.93 | 26.19 | 33.93 | 56.58 | 49.42 |
| SMR_VSA6 | 0.00 | 33.33 | 66.65 | 33.33 | 33.33 | 0.00 | 0.00 | 0.00 | 33.33 | 0.00 | 0.00 | 0.00 | 33.33 | 99.98 | 99.98 |
| SMR_VSA7 | 5.34 | 5.34 | 2.86 | 2.93 | 2.72 | 2.79 | 3.05 | 4.87 | 2.72 | 2.79 | 3.26 | 4.87 | 5.10 | 5.26 | 5.30 |
| std_dim1 | 2.33 | 1.44 | 1.54 | 1.60 | 1.49 | 1.48 | 1.47 | 1.36 | 1.49 | 1.48 | 1.66 | 1.87 | 2.81 | 2.14 | 2.24 |
| std_dim2 | 1.93 | 1.07 | 1.19 | 1.19 | 1.06 | 1.04 | 0.97 | 1.11 | 1.04 | 1.03 | 0.61 | 0.69 | 1.85 | 1.12 | 1.25 |
| std_dim3 | 109.48 | 114.37 | 99.10 | 119.33 | 99.10 | 119.33 | 128.61 | 131.69 | 99.10 | 119.33 | 140.79 | 143.87 | 138.94 | 149.90 | 162.08 |
| TPSA | 0.28 | 0.38 | 0.50 | 0.48 | 0.50 | 0.48 | 0.50 | 0.40 | 0.52 | 0.50 | 0.48 | 0.39 | 0.26 | 0.32 | 0.31 |
| VAdjEq | 6.73 | 5.95 | 5.32 | 5.39 | 5.32 | 5.39 | 5.32 | 5.86 | 5.25 | 5.32 | 5.52 | 6.00 | 6.83 | 6.25 | 6.36 |
| VAdjMa | 4.04 | 3.74 | 2.89 | 2.94 | 2.89 | 2.94 | 2.92 | 3.59 | 2.87 | 2.92 | 2.99 | 3.57 | 4.02 | 3.80 | 3.78 |
| VDistEq | 10.83 | 9.40 | 8.23 | 8.38 | 8.23 | 8.38 | 8.22 | 9.20 | 8.06 | 8.22 | 8.52 | 9.42 | 11.09 | 10.06 | 10.23 |
| VDistMa | 585.52 | 376.11 | 254.09 | 264.12 | 241.27 | 251.30 | 252.38 | 348.57 | 236.86 | 246.89 | 267.17 | 363.36 | 646.44 | 481.26 | 496.06 |
| vdw_area | 831.13 | 479.68 | 316.32 | 324.84 | 295.03 | 303.55 | 307.07 | 449.27 | 291.89 | 300.41 | 339.21 | 481.41 | 905.08 | 605.80 | 637.94 |
| vdw_vol | 590.88 | 345.25 | 230.50 | 235.88 | 214.75 | 222.13 | 223.25 | 325.25 | 213.00 | 217.88 | 242.88 | 342.88 | 639.75 | 442.25 | 459.63 |
| vol | 624.40 | 389.56 | 270.75 | 278.63 | 254.48 | 258.30 | 259.48 | 364.80 | 251.15 | 253.21 | 275.08 | 381.67 | 677.53 | 503.94 | 525.07 |
| VSA | 43.51 | 62.45 | 29.64 | 29.64 | 29.64 | 29.64 | 21.75 | 35.32 | 29.64 | 29.64 | 13.87 | 27.44 | 43.51 | 76.02 | 68.14 |
| vsa_acc | 0.00 | 0.00 | 0.00 | 0.00 | 0.00 | 0.00 | 0.00 | 0.00 | 0.00 | 0.00 | 0.00 | 0.00 | 0.00 | 0.00 | 0.00 |
| vsa_acid | 11.37 | 0.00 | 0.00 | 0.00 | 0.00 | 0.00 | 0.00 | 0.00 | 0.00 | 0.00 | 23.43 | 11.37 | 11.37 | 0.00 | 11.37 |
| vsa_base | 11.37 | 5.68 | 5.68 | 5.68 | 5.68 | 5.68 | 17.74 | 5.68 | 5.68 | 5.68 | 23.43 | 11.37 | 11.37 | 5.68 | 11.37 |
| vsa_don | 398.96 | 214.02 | 127.63 | 120.28 | 116.94 | 109.59 | 104.47 | 185.72 | 111.82 | 104.47 | 75.06 | 156.31 | 429.48 | 273.95 | 244.54 |
| vsa_hyd | 106.32 | 80.20 | 43.34 | 43.34 | 43.34 | 43.34 | 41.58 | 54.53 | 43.34 | 43.34 | 84.91 | 97.86 | 115.07 | 106.17 | 149.50 |
| vsa_other | 54.87 | 68.14 | 62.45 | 76.02 | 62.45 | 76.02 | 80.20 | 81.70 | 62.45 | 76.02 | 77.99 | 79.50 | 68.44 | 81.70 | 79.50 |
| vsa_pol | 0.00 | 0.00 | 0.00 | 0.00 | 0.00 | 0.00 | 0.00 | 0.00 | 0.00 | 0.00 | 0.00 | 0.00 | 0.00 | 0.00 | 0.00 |
| vsurf_A | 0.00 | 0.00 | 0.00 | 0.00 | 0.00 | 0.00 | 0.00 | 0.00 | 0.00 | 0.00 | 0.00 | 0.00 | 0.00 | 0.00 | 0.00 |
| vsurf_CP | 0.00 | 0.00 | 0.00 | 0.00 | 0.00 | 0.00 | 0.00 | 0.00 | 0.00 | 0.00 | 0.00 | 0.00 | 0.00 | 0.00 | 0.00 |
| vsurf_CW1 | 0.00 | 0.00 | 0.00 | 0.00 | 0.00 | 0.00 | 0.00 | 0.00 | 0.00 | 0.00 | 0.00 | 0.00 | 0.00 | 0.00 | 0.00 |
| vsurf_CW2 | 0.00 | 0.00 | 0.00 | 0.00 | 0.00 | 0.00 | 0.00 | 0.00 | 0.00 | 0.00 | 0.00 | 0.00 | 0.00 | 0.00 | 0.00 |
| vsurf_CW3 | 0.00 | 0.00 | 0.00 | 0.00 | 0.00 | 0.00 | 0.00 | 0.00 | 0.00 | 0.00 | 0.00 | 0.00 | 0.00 | 0.00 | 0.00 |
| vsurf_CW4 | 0.00 | 0.00 | 0.00 | 0.00 | 0.00 | 0.00 | 0.00 | 0.00 | 0.00 | 0.00 | 0.00 | 0.00 | 0.00 | 0.00 | 0.00 |
| vsurf_CW5 | 0.00 | 0.00 | 0.00 | 0.00 | 0.00 | 0.00 | 0.00 | 0.00 | 0.00 | 0.00 | 0.00 | 0.00 | 0.00 | 0.00 | 0.00 |
| vsurf_CW6 | 0.00 | 0.00 | 0.00 | 0.00 | 0.00 | 0.00 | 0.00 | 0.00 | 0.00 | 0.00 | 0.00 | 0.00 | 0.00 | 0.00 | 0.00 |
| vsurf_CW7 | 0.00 | 0.00 | 0.00 | 0.00 | 0.00 | 0.00 | 0.00 | 0.00 | 0.00 | 0.00 | 0.00 | 0.00 | 0.00 | 0.00 | 0.00 |
| vsurf_CW8 | 0.00 | 0.00 | 0.00 | 0.00 | 0.00 | 0.00 | 0.00 | 0.00 | 0.00 | 0.00 | 0.00 | 0.00 | 0.00 | 0.00 | 0.00 |
| vsurf_D1 | 0.00 | 0.00 | 0.00 | 0.00 | 0.00 | 0.00 | 0.00 | 0.00 | 0.00 | 0.00 | 0.00 | 0.00 | 0.00 | 0.00 | 0.00 |
| vsurf_D2 | 0.00 | 0.00 | 0.00 | 0.00 | 0.00 | 0.00 | 0.00 | 0.00 | 0.00 | 0.00 | 0.00 | 0.00 | 0.00 | 0.00 | 0.00 |
| vsurf_D3 | 0.00 | 0.00 | 0.00 | 0.00 | 0.00 | 0.00 | 0.00 | 0.00 | 0.00 | 0.00 | 0.00 | 0.00 | 0.00 | 0.00 | 0.00 |
| vsurf_D4 | 0.00 | 0.00 | 0.00 | 0.00 | 0.00 | 0.00 | 0.00 | 0.00 | 0.00 | 0.00 | 0.00 | 0.00 | 0.00 | 0.00 | 0.00 |
| vsurf_D5 | 0.00 | 0.00 | 0.00 | 0.00 | 0.00 | 0.00 | 0.00 | 0.00 | 0.00 | 0.00 | 0.00 | 0.00 | 0.00 | 0.00 | 0.00 |
| vsurf_D6 | 0.00 | 0.00 | 0.00 | 0.00 | 0.00 | 0.00 | 0.00 | 0.00 | 0.00 | 0.00 | 0.00 | 0.00 | 0.00 | 0.00 | 0.00 |
| vsurf_D7 | 0.00 | 0.00 | 0.00 | 0.00 | 0.00 | 0.00 | 0.00 | 0.00 | 0.00 | 0.00 | 0.00 | 0.00 | 0.00 | 0.00 | 0.00 |
| vsurf_D8 | 0.00 | 0.00 | 0.00 | 0.00 | 0.00 | 0.00 | 0.00 | 0.00 | 0.00 | 0.00 | 0.00 | 0.00 | 0.00 | 0.00 | 0.00 |
| vsurf_DD12 | 0.00 | 0.00 | 0.00 | 0.00 | 0.00 | 0.00 | 0.00 | 0.00 | 0.00 | 0.00 | 0.00 | 0.00 | 0.00 | 0.00 | 0.00 |
| vsurf_DD13 | 0.00 | 0.00 | 0.00 | 0.00 | 0.00 | 0.00 | 0.00 | 0.00 | 0.00 | 0.00 | 0.00 | 0.00 | 0.00 | 0.00 | 0.00 |
| vsurf_DD23 | 0.00 | 0.00 | 0.00 | 0.00 | 0.00 | 0.00 | 0.00 | 0.00 | 0.00 | 0.00 | 0.00 | 0.00 | 0.00 | 0.00 | 0.00 |
| vsurf_DW12 | 0.00 | 0.00 | 0.00 | 0.00 | 0.00 | 0.00 | 0.00 | 0.00 | 0.00 | 0.00 | 0.00 | 0.00 | 0.00 | 0.00 | 0.00 |
| vsurf_DW13 | 0.00 | 0.00 | 0.00 | 0.00 | 0.00 | 0.00 | 0.00 | 0.00 | 0.00 | 0.00 | 0.00 | 0.00 | 0.00 | 0.00 | 0.00 |
| vsurf_DW23 | 0.00 | 0.00 | 0.00 | 0.00 | 0.00 | 0.00 | 0.00 | 0.00 | 0.00 | 0.00 | 0.00 | 0.00 | 0.00 | 0.00 | 0.00 |
| vsurf_EDmin1 | 0.00 | 0.00 | 0.00 | 0.00 | 0.00 | 0.00 | 0.00 | 0.00 | 0.00 | 0.00 | 0.00 | 0.00 | 0.00 | 0.00 | 0.00 |
| vsurf_EDmin2 | 0.00 | 0.00 | 0.00 | 0.00 | 0.00 | 0.00 | 0.00 | 0.00 | 0.00 | 0.00 | 0.00 | 0.00 | 0.00 | 0.00 | 0.00 |
| vsurf_EDmin3 | 0.00 | 0.00 | 0.00 | 0.00 | 0.00 | 0.00 | 0.00 | 0.00 | 0.00 | 0.00 | 0.00 | 0.00 | 0.00 | 0.00 | 0.00 |
| vsurf_EWmin1 | 0.00 | 0.00 | 0.00 | 0.00 | 0.00 | 0.00 | 0.00 | 0.00 | 0.00 | 0.00 | 0.00 | 0.00 | 0.00 | 0.00 | 0.00 |
| vsurf_EWmin2 | 0.00 | 0.00 | 0.00 | 0.00 | 0.00 | 0.00 | 0.00 | 0.00 | 0.00 | 0.00 | 0.00 | 0.00 | 0.00 | 0.00 | 0.00 |
| vsurf_EWmin3 | 0.00 | 0.00 | 0.00 | 0.00 | 0.00 | 0.00 | 0.00 | 0.00 | 0.00 | 0.00 | 0.00 | 0.00 | 0.00 | 0.00 | 0.00 |
| vsurf_G | 0.00 | 0.00 | 0.00 | 0.00 | 0.00 | 0.00 | 0.00 | 0.00 | 0.00 | 0.00 | 0.00 | 0.00 | 0.00 | 0.00 | 0.00 |
| vsurf_HB1 | 0.00 | 0.00 | 0.00 | 0.00 | 0.00 | 0.00 | 0.00 | 0.00 | 0.00 | 0.00 | 0.00 | 0.00 | 0.00 | 0.00 | 0.00 |
| vsurf_HB2 | 0.00 | 0.00 | 0.00 | 0.00 | 0.00 | 0.00 | 0.00 | 0.00 | 0.00 | 0.00 | 0.00 | 0.00 | 0.00 | 0.00 | 0.00 |
| vsurf_HB3 | 0.00 | 0.00 | 0.00 | 0.00 | 0.00 | 0.00 | 0.00 | 0.00 | 0.00 | 0.00 | 0.00 | 0.00 | 0.00 | 0.00 | 0.00 |
| vsurf_HB4 | 0.00 | 0.00 | 0.00 | 0.00 | 0.00 | 0.00 | 0.00 | 0.00 | 0.00 | 0.00 | 0.00 | 0.00 | 0.00 | 0.00 | 0.00 |
| vsurf_HB5 | 0.00 | 0.00 | 0.00 | 0.00 | 0.00 | 0.00 | 0.00 | 0.00 | 0.00 | 0.00 | 0.00 | 0.00 | 0.00 | 0.00 | 0.00 |
| vsurf_HB6 | 0.00 | 0.00 | 0.00 | 0.00 | 0.00 | 0.00 | 0.00 | 0.00 | 0.00 | 0.00 | 0.00 | 0.00 | 0.00 | 0.00 | 0.00 |
| vsurf_HB7 | 0.00 | 0.00 | 0.00 | 0.00 | 0.00 | 0.00 | 0.00 | 0.00 | 0.00 | 0.00 | 0.00 | 0.00 | 0.00 | 0.00 | 0.00 |
| vsurf_HB8 | 0.00 | 0.00 | 0.00 | 0.00 | 0.00 | 0.00 | 0.00 | 0.00 | 0.00 | 0.00 | 0.00 | 0.00 | 0.00 | 0.00 | 0.00 |
| vsurf_HL1 | 0.00 | 0.00 | 0.00 | 0.00 | 0.00 | 0.00 | 0.00 | 0.00 | 0.00 | 0.00 | 0.00 | 0.00 | 0.00 | 0.00 | 0.00 |
| vsurf_HL2 | 0.00 | 0.00 | 0.00 | 0.00 | 0.00 | 0.00 | 0.00 | 0.00 | 0.00 | 0.00 | 0.00 | 0.00 | 0.00 | 0.00 | 0.00 |
| vsurf_ID1 | 0.00 | 0.00 | 0.00 | 0.00 | 0.00 | 0.00 | 0.00 | 0.00 | 0.00 | 0.00 | 0.00 | 0.00 | 0.00 | 0.00 | 0.00 |
| vsurf_ID2 | 0.00 | 0.00 | 0.00 | 0.00 | 0.00 | 0.00 | 0.00 | 0.00 | 0.00 | 0.00 | 0.00 | 0.00 | 0.00 | 0.00 | 0.00 |
| vsurf_ID3 | 0.00 | 0.00 | 0.00 | 0.00 | 0.00 | 0.00 | 0.00 | 0.00 | 0.00 | 0.00 | 0.00 | 0.00 | 0.00 | 0.00 | 0.00 |
| vsurf_ID4 | 0.00 | 0.00 | 0.00 | 0.00 | 0.00 | 0.00 | 0.00 | 0.00 | 0.00 | 0.00 | 0.00 | 0.00 | 0.00 | 0.00 | 0.00 |
| vsurf_ID5 | 0.00 | 0.00 | 0.00 | 0.00 | 0.00 | 0.00 | 0.00 | 0.00 | 0.00 | 0.00 | 0.00 | 0.00 | 0.00 | 0.00 | 0.00 |
| vsurf_ID6 | 0.00 | 0.00 | 0.00 | 0.00 | 0.00 | 0.00 | 0.00 | 0.00 | 0.00 | 0.00 | 0.00 | 0.00 | 0.00 | 0.00 | 0.00 |
| vsurf_ID7 | 0.00 | 0.00 | 0.00 | 0.00 | 0.00 | 0.00 | 0.00 | 0.00 | 0.00 | 0.00 | 0.00 | 0.00 | 0.00 | 0.00 | 0.00 |
| vsurf_ID8 | 0.00 | 0.00 | 0.00 | 0.00 | 0.00 | 0.00 | 0.00 | 0.00 | 0.00 | 0.00 | 0.00 | 0.00 | 0.00 | 0.00 | 0.00 |
| vsurf_IW1 | 0.00 | 0.00 | 0.00 | 0.00 | 0.00 | 0.00 | 0.00 | 0.00 | 0.00 | 0.00 | 0.00 | 0.00 | 0.00 | 0.00 | 0.00 |
| vsurf_IW2 | 0.00 | 0.00 | 0.00 | 0.00 | 0.00 | 0.00 | 0.00 | 0.00 | 0.00 | 0.00 | 0.00 | 0.00 | 0.00 | 0.00 | 0.00 |
| vsurf_IW3 | 0.00 | 0.00 | 0.00 | 0.00 | 0.00 | 0.00 | 0.00 | 0.00 | 0.00 | 0.00 | 0.00 | 0.00 | 0.00 | 0.00 | 0.00 |
| vsurf_IW4 | 0.00 | 0.00 | 0.00 | 0.00 | 0.00 | 0.00 | 0.00 | 0.00 | 0.00 | 0.00 | 0.00 | 0.00 | 0.00 | 0.00 | 0.00 |
| vsurf_IW5 | 0.00 | 0.00 | 0.00 | 0.00 | 0.00 | 0.00 | 0.00 | 0.00 | 0.00 | 0.00 | 0.00 | 0.00 | 0.00 | 0.00 | 0.00 |
| vsurf_IW6 | 0.00 | 0.00 | 0.00 | 0.00 | 0.00 | 0.00 | 0.00 | 0.00 | 0.00 | 0.00 | 0.00 | 0.00 | 0.00 | 0.00 | 0.00 |
| vsurf_IW7 | 0.00 | 0.00 | 0.00 | 0.00 | 0.00 | 0.00 | 0.00 | 0.00 | 0.00 | 0.00 | 0.00 | 0.00 | 0.00 | 0.00 | 0.00 |
| vsurf_IW8 | 0.00 | 0.00 | 0.00 | 0.00 | 0.00 | 0.00 | 0.00 | 0.00 | 0.00 | 0.00 | 0.00 | 0.00 | 0.00 | 0.00 | 0.00 |
| vsurf_R | 0.00 | 0.00 | 0.00 | 0.00 | 0.00 | 0.00 | 0.00 | 0.00 | 0.00 | 0.00 | 0.00 | 0.00 | 0.00 | 0.00 | 0.00 |
| vsurf_S | 0.00 | 0.00 | 0.00 | 0.00 | 0.00 | 0.00 | 0.00 | 0.00 | 0.00 | 0.00 | 0.00 | 0.00 | 0.00 | 0.00 | 0.00 |
| vsurf_V | 0.00 | 0.00 | 0.00 | 0.00 | 0.00 | 0.00 | 0.00 | 0.00 | 0.00 | 0.00 | 0.00 | 0.00 | 0.00 | 0.00 | 0.00 |
| vsurf_W1 | 0.00 | 0.00 | 0.00 | 0.00 | 0.00 | 0.00 | 0.00 | 0.00 | 0.00 | 0.00 | 0.00 | 0.00 | 0.00 | 0.00 | 0.00 |
| vsurf_W2 | 0.00 | 0.00 | 0.00 | 0.00 | 0.00 | 0.00 | 0.00 | 0.00 | 0.00 | 0.00 | 0.00 | 0.00 | 0.00 | 0.00 | 0.00 |
| vsurf_W3 | 0.00 | 0.00 | 0.00 | 0.00 | 0.00 | 0.00 | 0.00 | 0.00 | 0.00 | 0.00 | 0.00 | 0.00 | 0.00 | 0.00 | 0.00 |
| vsurf_W4 | 0.00 | 0.00 | 0.00 | 0.00 | 0.00 | 0.00 | 0.00 | 0.00 | 0.00 | 0.00 | 0.00 | 0.00 | 0.00 | 0.00 | 0.00 |
| vsurf_W5 | 0.00 | 0.00 | 0.00 | 0.00 | 0.00 | 0.00 | 0.00 | 0.00 | 0.00 | 0.00 | 0.00 | 0.00 | 0.00 | 0.00 | 0.00 |
| vsurf_W6 | 0.00 | 0.00 | 0.00 | 0.00 | 0.00 | 0.00 | 0.00 | 0.00 | 0.00 | 0.00 | 0.00 | 0.00 | 0.00 | 0.00 | 0.00 |
| vsurf_W7 | 0.00 | 0.00 | 0.00 | 0.00 | 0.00 | 0.00 | 0.00 | 0.00 | 0.00 | 0.00 | 0.00 | 0.00 | 0.00 | 0.00 | 0.00 |
| vsurf_W8 | 0.00 | 0.00 | 0.00 | 0.00 | 0.00 | 0.00 | 0.00 | 0.00 | 0.00 | 0.00 | 0.00 | 0.00 | 0.00 | 0.00 | 0.00 |
| vsurf_Wp1 | 0.00 | 0.00 | 0.00 | 0.00 | 0.00 | 0.00 | 0.00 | 0.00 | 0.00 | 0.00 | 0.00 | 0.00 | 0.00 | 0.00 | 0.00 |
| vsurf_Wp2 | 0.00 | 0.00 | 0.00 | 0.00 | 0.00 | 0.00 | 0.00 | 0.00 | 0.00 | 0.00 | 0.00 | 0.00 | 0.00 | 0.00 | 0.00 |
| vsurf_Wp3 | 0.00 | 0.00 | 0.00 | 0.00 | 0.00 | 0.00 | 0.00 | 0.00 | 0.00 | 0.00 | 0.00 | 0.00 | 0.00 | 0.00 | 0.00 |
| vsurf_Wp4 | 0.00 | 0.00 | 0.00 | 0.00 | 0.00 | 0.00 | 0.00 | 0.00 | 0.00 | 0.00 | 0.00 | 0.00 | 0.00 | 0.00 | 0.00 |
| vsurf_Wp5 | 0.00 | 0.00 | 0.00 | 0.00 | 0.00 | 0.00 | 0.00 | 0.00 | 0.00 | 0.00 | 0.00 | 0.00 | 0.00 | 0.00 | 0.00 |
| vsurf_Wp6 | 0.00 | 0.00 | 0.00 | 0.00 | 0.00 | 0.00 | 0.00 | 0.00 | 0.00 | 0.00 | 0.00 | 0.00 | 0.00 | 0.00 | 0.00 |
| vsurf_Wp7 | 0.00 | 0.00 | 0.00 | 0.00 | 0.00 | 0.00 | 0.00 | 0.00 | 0.00 | 0.00 | 0.00 | 0.00 | 0.00 | 0.00 | 0.00 |
| vsurf_Wp8 | 626.67 | 401.35 | 274.25 | 290.25 | 278.21 | 294.21 | 275.24 | 379.34 | 260.22 | 276.22 | 300.27 | 404.38 | 688.74 | 505.45 | 530.49 |
| Weight | 8343.00 | 2478.00 | 652.00 | 752.00 | 652.00 | 752.00 | 660.00 | 1972.00 | 568.00 | 660.00 | 853.00 | 2269.00 | 9959.00 | 4165.00 | 4633.00 |
| weinerPath | 84.00 | 45.00 | 34.00 | 36.00 | 34.00 | 36.00 | 33.00 | 46.00 | 31.00 | 33.00 | 38.00 | 51.00 | 92.00 | 57.00 | 62.00 |
| weinerPol | 256.00 | 148.00 | 100.00 | 104.00 | 100.00 | 104.00 | 98.00 | 140.00 | 94.00 | 98.00 | 114.00 | 156.00 | 276.00 | 182.00 | 198.00 |
| zagreb |  |  |  |  |  |  |  |  |  |  |  |  |  |  |  |
