# Supplementary material for: An Integrated In Silico Approach to Design Specific Inhibitors Targeting Human Poly(A)-Specific Ribonuclease
Source: PLoS One. 2012 Dec 6;7(12):e51113. doi: 10.1371/journal.pone.0051113 (PMC3516499; doi:10.1371/journal.pone.0051113)
Supplement: Table S3 — Drug likeness properties of our previously reported nucleoside analog inhibitors of PARN, including the consensus score of drug likeness, a toxicity measure and an ease-of-synthesis approximation. (DOCX) [file pone.0051113.s008.docx]

**Table S3**

| **Compound id** | **ki** | **weight** | **TPSA** | **DON** | **ACC** | **logP** | **logS** | **toxic** | **rsynth** | **Drug Likeness** |
| --- | --- | --- | --- | --- | --- | --- | --- | --- | --- | --- |
| **U1** | 19 | 276.22 | 119.33 | 4 | 6 | -2.17 | -0.2 | no | 100 | 100 |
| **FU1** | 98 | 294.21 | 119.33 | 4 | 6 | -1.87 | -0.58 | no | 100 | 100 |
| **T1** | 135 | 290.25 | 119.33 | 4 | 6 | -1.78 | -0.21 | no | 100 | 100 |
| **A6** | 210 | 379.34 | 131.69 | 4 | 7 | -0.46 | -2.45 | no | 100 | 100 |
| **A2** | 510 | 404.38 | 143.87 | 5 | 7 | -0.46 | -3.51 | no | 100 | 100 |
| **C6** | 645 | 275.24 | 128.61 | 4 | 6 | -1.93 | -0.4 | no | 100 | 100 |
| **A4** | 767 | **688.74** | 138.94 | 3 | 7 | 5.44 | -9.87 | no | 100 | 0 |
| **A5** | 868 | **505.45** | 149.9 | 1 | 7 | 1.25 | -4.3 | no | 100 | 0 |
| **A1** | 872 | **530.49** | 162.08 | 2 | 7 | 1.26 | -5.37 | no | 100 | 0 |
| **A3** | 992 | **626.67** | 109.48 | 2 | 6 | 6.23 | -10.47 | no | 100 | 0 |
| **T2** | 1000 | 274.25 | 99.1 | 3 | 5 | -0.75 | -0.74 | no | 100 | 100 |
| **U4** | 1000 | 278.21 | 99.1 | 3 | 5 | -0.85 | -1.11 | no | 100 | 100 |
| **U3** | 1000 | 260.22 | 99.1 | 3 | 5 | -1.14 | -0.73 | no | 100 | 100 |
| **C2** | 1000 | 300.27 | 140.79 | 5 | 6 | -2.13 | -1.26 | **yes** | 100 | 0 |
| **A7** | 1000 | 401.35 | 114.37 | 1 | 6 | 1.47 | -4.37 | no | **75.86** | 0 |
