# Supplementary material for: An Integrated In Silico Approach to Design Specific Inhibitors Targeting Human Poly(A)-Specific Ribonuclease
Source: PLoS One. 2012 Dec 6;7(12):e51113. doi: 10.1371/journal.pone.0051113 (PMC3516499; doi:10.1371/journal.pone.0051113)
Supplement: Table S4 — Poly(A) and DNP-poly(A) polymer properties prediction values. (DOCX) [file pone.0051113.s009.docx]

**Table S4**

| **POLYMER PROPERTIES** | **RNA** | **RNA-DNP** |
| --- | --- | --- |
| Molecular Weight of Repeat Unit | 328.2010 g/mol | 478.2940 g/mol |
| Length of Extended Repeat Unit | 7.3730 A | 7.3191 A |
| Amorphous Molar Volume | 238.4419 cc/mol | 336.1792 cc/mol |
| van der Waals Volume | 158.6172 cc/mol | 224.2999 cc/mol |
| Amorphous Density | 1.3764 g/cc | 1.4227 g/cc |
| Cohesive Energy (Fedors) | 141045.5873 J/mol | 170896.5510 J/mol |
| Cohesive Energy (van Krevelen) | 126411.1677 J/mol | 132019.4377 J/mol |
| Solubility Parameter (Fedors) | 24.3214 (J/cc)**0.5 | 22.5466 (J/cc)**0.5 |
| Solubility Parameter (van Krevelen) | 23.0251 (J/cc)**0.5 | 19.8168 (J/cc)**0.5 |
| Glass Transition Temperature | 466.2313 K | 452.9686 K |
| Volumetric Thermal Expansion | 212.1696 10**-6 1/K | 217.9783 10**-6 1/K |
| Heat Capacity | 372.2640 J/molK | 518.3154 J/molK |
| Heat Capacity Jump at Tg | 92.4633 J/molK | 109.5238 J/molK |
| Surface Tension (Fedors) | 52.8501 dyn/cm | 47.7716 dyn/cm |
| Surface Tension (van Krevelen) | 49.1281 dyn/cm | 40.2197 dyn/cm |
| Surface Tension (molar parachor) | 47.3015 dyn/cm | 48.5459 dyn/cm |
| Refractive Index | 1.6115 | 1.6021 |
| Molar Refraction | 82.8273 cc/mol | 115.3269 cc/mol |
| Dielectric Constant | 3.9600 | 3.7949 |
| Effective Dipole Moment | 1.3145 debyes | 1.5075 debyes |
| Molar Parachor | 625.3184 | 887.3786 |
| Molar Polarization | 118.4206 cc/mole | 162.1405 cc/mole |
| Volume Resistivity (log10) | 15.0800 ohm cm | 15.4102 ohm cm |
| Magnetic Susceptibility | 0.5372 1e-6 cc/g | 0.5321 1e-6 cc/g |
| Entanglement Molecular Weight | 12556.0394 g/mol | 25300.7697 g/mol |
| Steric Hindrance Parameter | 2.7461 | 2.9394 |
| Characteristic Ratio | 15.0822 | 17.2807 |
| Molar Stiffness | 90.0774 | 118.4695 |
| Critical Molecular Weight | 29784.0048 g/mol | 44899.3174 g/mol |
| Activation Energy for Viscous Flow | 61091.9648 J/mol | 55260.7785 J/mol |
| Thermal Conductivity | 0.1599 J/Kms | 0.1518 J/Kms |
| Permeability to O2 | 1.1779 Dow Units | 78.4881 Dow Units |
| Permeability to N2 | 0.2041 Dow Units | 19.0580 Dow Units |
| Permeability to CO2 | 3.4799 Dow Units | 337.3143 Dow Units |
| Activation Energy of Decomposition | 278.7856 kJ/mol | 288.2287 kJ/mol |
| Temperature of Initial Decomposition | 631.6071 K | 640.1059 K |
| Temperature of Half Decomposition | 701.7856 K | 711.2287 K |
| Temperature of Maximum Decomposition | 701.7856 K | 711.2287 K |
| Molar Thermal Decomposition Function | 230.3267 kgK/mol | 340.1764 kgK/mol |
| Dispersion Component of Molar Attraction | 4256.3847 | 6072.7879 |
