# Supplementary material for: An Integrated In Silico Approach to Design Specific Inhibitors Targeting Human Poly(A)-Specific Ribonuclease
Source: PLoS One. 2012 Dec 6;7(12):e51113. doi: 10.1371/journal.pone.0051113 (PMC3516499; doi:10.1371/journal.pone.0051113)
Supplement: Table S5 — Summary of the compounds and their corresponding inhibition constants used in our statistical analysis and pharmacophore design. (DOCX) [file pone.0051113.s010.docx]

**Table S5**

| Compound | Structural formula | *K*_i_, μM |  | Compound | Structural formula | *K*_i_, μM |
| --- | --- | --- | --- | --- | --- | --- |
| Α1 | 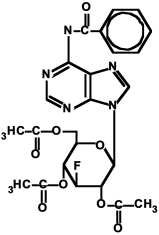 | 873 ± 92 |  | T2 | 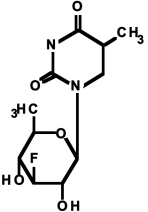 | >1000 |
| Α2 | 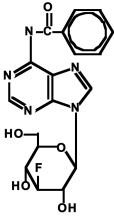 | 510 ± 52 |  | U1 | 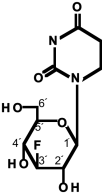 | 19 ± 5 |
| Α3 | 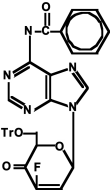 | 992 ± 89 |  | U2 (FU1) | 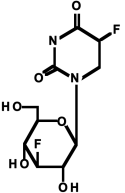 | 98 ± 12 |
| Α4 | 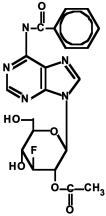 | 767 ± 59 |  | U3 | 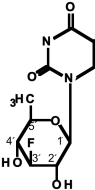 | >1000 |
| Α5 | 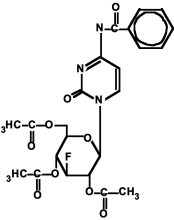 | 868 ± 67 |  | U4 (FU2) | 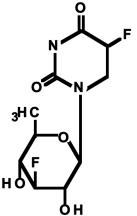 | >1000 |
| Α6 | 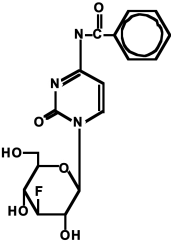 | 210 ± 45 |  | C6 | 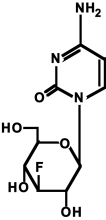 | 645 ± 37 |
| Α7 | 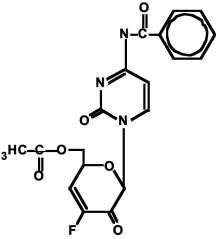 | 1112 ± 89 |  | C2 | 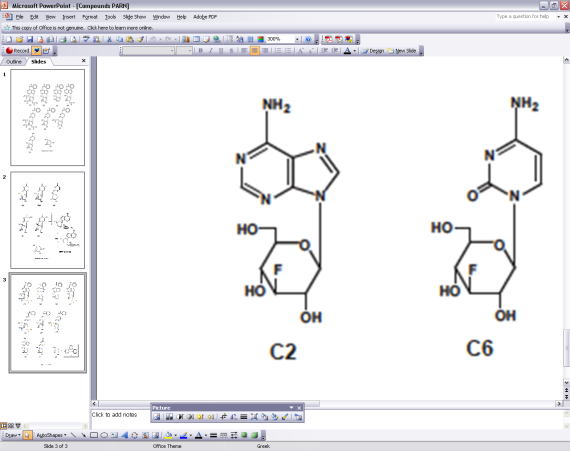 | >1000 |
| T1 | 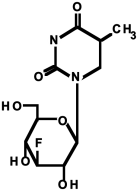 | 135 ± 18 |  | DNP | 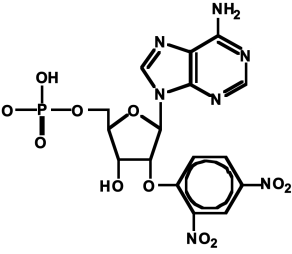 | 98 ± 7 |
