# Supplementary material for: An Integrated In Silico Approach to Design Specific Inhibitors Targeting Human Poly(A)-Specific Ribonuclease
Source: PLoS One. 2012 Dec 6;7(12):e51113. doi: 10.1371/journal.pone.0051113 (PMC3516499; doi:10.1371/journal.pone.0051113)
Supplement: Table S6 — Summary of the inhibitor compounds of table S5 and their corresponding interaction energies with the catalytic site of PARN. Interaction energies (Int. E.) are represented in Kcal/mole units and have been calculated using the potential energy module of MOE. (DOCX) [file pone.0051113.s011.docx]

**Table S6**

| Compound | *Int. E.* |  | Compound | *Int. E.* |
| --- | --- | --- | --- | --- |
| Α1 | -2.817 |  | T2 | 0.311 |
| Α2 | -8.230 |  | U1 | -41.359 |
| Α3 | -2.612 |  | U2 (FU1) | -20.985 |
| Α4 | -3.092 |  | U3 | 0.844 |
| Α5 | 2.911 |  | U4 (FU2) | 0.991 |
| Α6 | -14.671 |  | C6 | -1.590 |
| Α7 | -0.505 |  | C2 | 1.188 |
| T1 | -17.222 |  | DNP (A) | -19.933 |
