# Supplementary material for: An Integrated In Silico Approach to Design Specific Inhibitors Targeting Human Poly(A)-Specific Ribonuclease
Source: PLoS One. 2012 Dec 6;7(12):e51113. doi: 10.1371/journal.pone.0051113 (PMC3516499; doi:10.1371/journal.pone.0051113)
Supplement: Text S1 — Statistical evaluation of structure activity relationships. (DOCX) [file pone.0051113.s012.docx]

**Text S1**

Following the in-depth structure-based analysis of the nuclease domain of PARN, we used a ligand-based and custom developed statistical structure / activity statistical analysis, in an effort to pinpoint and identify the features of PARN’s catalytic site that are crucial for the recognition of potent inhibitor compounds. Working on a combined complex-based receptor-ligand approach, firstly the compound moieties that enhance the inhibition of PARN are identified and then PARN’s machinery for efficient recognition of those moieties is used to design a 3D pharmacophore and propose new inhibitors.

In order to obtain a qualitative estimate of the relationships between the molecular structures of our inhibitors and their biological activities, we estimated the correlation relationships between the reported Ki activity and the molecular electronic properties measured. The molecular descriptors that were used for this study are summarized in Table S2.

Since our nucleoside analogs are a congeneric series of compounds (Table S5), their biological activities can be correlated either with respect to certain structural features (i.e. atomic, group, moiety) or molecular properties (i.e. lipophilicity, number of acceptor/donor atoms, electronic and steric properties), in an attempt to illuminate the fundamental molecular characteristics responsible for their potency.

We used a total of 330 molecular properties descriptors (Table S2) for our previously reported nucleoside analog compounds (Table S5). For each descriptor C and R^2^ correlation coefficients were measured to report on their correlation structure with Ki activity. Fig. S3A shows the hierarchical clustering with resampling of the 15 compounds when only descriptors highly correlated with Ki were selected, namely we considered only 84 descriptors with C>0.6 and R^2^>0.2 values (coloured red in Table S2). Two groups of compounds are estimated by pvclust algorithm, one of which is consisting solely of adenosine based compounds. The red rectangles shown in the plot indicate that both clusters are highly supported by the data with AU≥95% (a more analytical explanation of the algorithm can be found in Methods section). Especially for the adenosine based compounds cluster, the reported AU p-value equals 99% with standard error 0.001, rejecting the hypothesis that the cluster does not exist at a 0.01 significance level.

In order to better visualize and highlight the similarities in the data, we employed Principal Component Analysis (PCA) on a subgroup of 239 descriptors, i.e. only descriptors with non-zero values were considered ([36](#_ENREF_36)). We found that the two first principal components could explain the 92% of the variability in the data. Most descriptors appear to have PC score values around zero with few outliers. Fig. S3B shows the compounds’ loadings, which reveal how the variation in the measurements is aligned with the variation in the data. As can be seen, the adenosine based compounds’ loadings values, coloured in black, appear to increase in a linear fashion when the data is projected to the first and second principal components estimated (PC1, PC2). Compounds A7 and C2 have been omitted from this study, since they are considered to be outliers based on difficulty in chemical synthesis and toxicity predictions (Table S3).

When PC loadings data are projected onto the second and third principal components (PC2, PC3), the green group of compounds is appearing in the first quartile of the plot showing high homogeneity. Overall, variables, which mostly contribute to the PCs, are plotted around the borders of the plot. Both hierarchical clustering analysis and PCA identified similar patterns in the data, by separately grouping the adenosine and non adenosine based compounds. High scatter diversity was observed for the adenosine based compounds, over the non-adenosine cluster. However in +/- quadrant our statistical evaluation positioned only A2 and A6 compounds, which constitute the most potent adenosine based inhibitors of PARN (blue region). Strikingly, statistical analysis abides with strict Lipinsky drug-likeness criteria, where we observed a linear increase of PARN inhibition potency, with a decrease in molecular weight and the LogP of the adenosine based compounds (Fig. S3C).

Taken together, the adenosine based compound analysis has successfully correlated the observed kinetic activity of those molecules as a function of their calculated molecular descriptors. Therefore, we propose that our analysis can be used as a potential prediction tool for ligand-based drug design experiments of adenosine based inhibitors, which should be accordingly evaluated and screened prior to their cost- and time consuming *in vitro* evaluation.

Based on the above statistical information about PARN’s catalytic site we used 3D Pharmacophore design methods, which take into account both the three-dimensional structures and binding modes of receptors and inhibitors in order to identify regions that are favorable for a specific receptor-inhibitor interaction (18). The description of the receptor-inhibitor interaction pattern is determined by a correlation between the characteristic properties of the inhibitors and their biochemically determined enzymatic activity ([19](#_ENREF_41)).
